# Proteome and Acetyl-Proteome Profiling of *Camellia sinensis* cv. ‘Anji Baicha’ during Periodic Albinism Reveals Alterations in Photosynthetic and Secondary Metabolite Biosynthetic Pathways

**DOI:** 10.3389/fpls.2017.02104

**Published:** 2017-12-11

**Authors:** Yan-Xia Xu, Wei Chen, Chun-Lei Ma, Si-Yan Shen, Yan-Yan Zhou, Lian-Qi Zhou, Liang Chen

**Affiliations:** ^1^Key Laboratory of Tea Biology and Resources Utilization, Ministry of Agriculture, National Center for Tea Improvement, Tea Research Institute of the Chinese Academy of Agricultural Sciences, Hangzhou, China; ^2^Jingjie PTM Biolab (Hangzhou) Co., Ltd., Hangzhou, China

**Keywords:** acetylation, acetylome, albino, metabolism, PTM, proteomic, tea, overlap

## Abstract

Tea leaf color is not only important from an aesthetics standpoint but is also related to tea quality. To investigate the molecular mechanisms that determine tea leaf color, we examined *Camellia sinensis* cv. ‘Anji Baicha’ (an albino tea cultivar) by tandem mass tag isobaric labeling to generate a high-resolution proteome and acetyl-proteome atlas of three leaf developmental stages. We identified a total of 7,637 proteins and quantified 6,256; of these, 3,232 were classified as differentially accumulated proteins (DAPs). We also identified 3,161 lysine acetylation sites in 1,752 proteins and quantified 2,869 in 1,612 proteins. The acetylation levels at 468 sites were significantly altered across the three developmental stages during periodic albinism; the corresponding proteins were associated with a variety of biological processes. Interestingly, a large number of DAPs and acetylated proteins with increased/decreased acetylation were related to photosynthesis and secondary metabolite biosynthetic pathways, suggesting that the accumulation or acetylation level of these proteins regulates periodic albinism in ‘Anji Baicha.’ Additionally, overlap between succinylome and acetylome among three ‘Anji Baicha’ developmental stages were found. These data provide important insight into the mechanisms of leaf coloration in the tea plant. The mass spectrometry data have been deposited to Proteome X change via the PRIDE partner repository with the data set identifier PXD008134.

## Introduction

The tea plant [*Camellia sinensis* (L.) O. Kuntze] is widely consumed as a non-alcoholic beverage and is thus an economically important woody crop (Chen et al., 2007). As living standards have improved, the requirement for high-quality tea has increased worldwide. *C. sinensis* ‘Anji Baicha’ (also known as “Baiye 1” or “White Leaf 1”) is a cultivar with an albino phenotype that is used to produce high-quality green tea. Young ‘Anji Baicha’ shoots are yellow-green in early spring when the temperature is below 20°C. The leaves become white as they fully expand, and gradually return to green as the environmental temperature increases (Cheng et al., 1999; Li, 2002; Li et al., 2011, 2015a). Previous studies have reported a positive correlation between amino acid concentration and albinism as well as a negative correlation between tea polyphenol levels and albinism (Li et al., 1996; Du et al., 2006; Xiong et al., 2013). Thus, the quality of ‘Anji Baicha’ is higher when new shoots become albino due to their high amino acid and modest tea polyphenol contents.

Large-scale -omics technologies have been used to investigate the molecular mechanisms underlying diverse cellular processes. High-throughput comparative proteomic approaches can provide information about cellular processes since proteins drive cellular function. Thus, using proteomics to clarify the molecular basis of leaf albinism in ‘Anji Baicha’ can be useful for improving tea quality. Some studies have examined the physiological changes in chloroplast ultrastructure, chemical composition, and enzymatic activity that occur during periodic albinism (Li et al., 1996, 2011; Du et al., 2006; Xiong et al., 2013), with certain genes and proteins found to be differentially expressed or accumulated at different stages of leaf development (Li et al., 2011; Ma et al., 2012; Yuan et al., 2015). However, proteomic data on tea plants are lacking, especially with respect to post-translational modifications (PTMs) such as lysine acetylation.

Plants adjust their metabolism to environmental conditions, leading to changes in protein activity, accumulation, and PTM (Prabakaran et al., 2012). PTMs are known to regulate various processes, including DNA and protein-protein interactions, enzyme activation, and protein stability. Hundreds of PTMs have been identified to date including lysine acetylation, which is highly prevalent and reversible (Zhang et al., 2009; Wu et al., 2011). Gene expression is modulated by lysine acetylation through the antagonistic activities of lysine deacetylases (KDAC) and lysine acetyl transferases (KAT) (Kouzarides, 2000; Yang, 2004; Lee and Workman, 2007; Shahbazian and Grunstein, 2007; Choudhary et al., 2009). Non-histone protein acetylation has been recently reported (Liu et al., 2014; Pan et al., 2014; Mo et al., 2015). Multiple KDACs and KATs have been characterized in plants (Pandey et al., 2002; Hu et al., 2009; Nallamilli et al., 2014). For example, *Arabidopsis* lysine acetyl transferase AtGCN5/HAG1 was reported to modulate the expression of development-related and inducible genes (Servet et al., 2010); AtTAF1 has been shown to control the greening of seedlings (Benhamed et al., 2006); and AtHAC1 regulates flowering time (Deng et al., 2007). Additionally, rice alcohol dehydrogenase 1 and pyruvate decarboxylase 1 are two submergence-inducible proteins that are acetylated on histone H3 (Tsuji et al., 2006). Increased acetylation at H3K9 of rice flowering locus 1 is correlated with transcriptional activation and the regulation of floral transition under short-day conditions (Komiya et al., 2008; Zhou and Hu, 2010). Large-scale screens of acetylated sites (ASs) and acetylated proteins (APs) have been performed in *Arabidopsis* (Finkemeier et al., 2011; Tan et al., 2011; Wu et al., 2011; König et al., 2014), rice (Nallamilli et al., 2014; Xiong et al., 2016), soybean (Smith-Hammond et al., 2014b), pea (Smith-Hammond et al., 2014a), grape (Melo-Braga et al., 2012), strawberry (Fang et al., 2015), and wheat (Zhang et al., 2016).

To clarify the mechanism underlying the albescence of ‘Anji Baicha’ leaves, we used a proteomics approach in combination with antibody-based enrichment. We present here for the first time the ‘Anji Baicha’ comprehensive proteome during periodic albinism and the first quantitative acetyl-proteome profiling of *C. sinensis* cv. ‘Anji Baicha’ leaves. Our findings provide important information regarding the molecular basis of leaf color change in the tea plant, which may be useful for further improving the quality of ‘Anji Baicha’ tea leaves.

## Materials and methods

### Plant material

The ‘Anji Baicha’ [*C. sinensis* (L.) O. Kuntze] plants used in this study were 10 years old and were grown in the fields of the Tea Research Institute of the Chinese Academy of Agricultural Sciences in Hangzhou, China. A total of 30 healthy tea plants were used, with three biological replicates per sample. Healthy leaves were collected during the spring from April to June 2015 from 10 different tea plants per replicate (2 g fresh weight for per tea plant), and immediately frozen in liquid nitrogen and stored at −80°C until use.

### Determination of chlorophyll and carotenoid contents

The chlorophyll and carotenoid contents in extracts (acetone:ethanol:water = 4.5:4.5:1 [v/v/v]) were estimated as previously described (Lichtenthaler and Wellburn, 1983). Each extract contained 100 mg of leaves and was homogenized for 24 h.

### Determination of total polyphenol and free amino acid contents

Leaves were freeze-dried until they were completely dried. Powdered dry samples were used for determination of total polyphenol and free amino acid contents. Polyphenols were extracted and measured spectrophotometrically according to the Folin-Ciocalteu method (Zhou et al., 2008). Gallic acid was used as the standard. Free amino acid content was measured using a Saikamu automatic amino acid analyzer (Tan et al., 2014).

### Protein preparation and trypsin digestion

Protein was extracted using trichloroacetic acid (TCA) combined with acetone (Semane et al., 2010). ‘Anji Baicha’ leaves were first ground in liquid nitrogen into a powder, which was washed with 10% TCA dissolved in acetone to remove chlorophyll. After three washes with pre-chilled acetone, the sample was dissolved in lysis buffer containing 10 mM dithiothreitol (DTT), 8 M urea, 1% protease inhibitor cocktail, and 2 mM ethylene diamine tetraacetic acid (EDTA). After sonication for 3 min (23 bursts, 3 s per burst) in ice-cold water at a 35% maximum power, the protein was precipitated with 15% TCA added dropwise for 2 h at −20°C. The precipitate was collected by centrifugation at 5,000 × g for 10 min at 4°C and washed with cold acetone; the supernatant was redissolved in 100 mM tetraethylammonium bromide (TEAB) with 8 M urea (pH 8.0). Protein concentration was determined using the 2-D Quant kit (GE Healthcare, Little Chalfont, UK) according to the manufacturer's protocol. Eight mg proteins from each sample with three biological replicates were digested with trypsin-based method. The protein solution was reduced in 10 mM DTT for 1 h at 37°C and alkylated with 20 mM iodoacetamide for 45 min at room temperature in the dark. Sequencing grade modified trypsin (Promega) was added at trypsin-to-protein mass ratios of 1:50 for the overnight digestion and 1:100 for the subsequent 4-h digestion.

### Tandem mass tag (TMT) labeling and high-performance liquid chromatography (HPLC)

Proteome and acetylome profiling were carried out with three biological replicates. Peptides were desalted on a Strata X C18 SPE column (Phenomenex, Macclesfield, UK), vacuum dried, and reconstituted in 0.5 M TEAB using the 6-plex TMT kit (Thermo Fisher Scientific, Waltham, MA, USA) according to the manufacturer's protocol. Briefly, 1 U of TMT reagent (defined as the amount of reagent required to label 100 μg of protein) was thawed and reconstituted in 24 μl acetonitrile. The peptide mixture was then incubated for 2 h at room temperature, pooled, desalted, and vacuum-dried. The TMT-labeled peptides were fractionated by high-pH reversed-phase HPLC using an 300 Extend C18 column (5-μm particle size, 4.6 mm inner diameter, 250 mm length) (Agilent Technologies, Santa Clara, CA, USA). Briefly, the peptides were first separated into 80 fractions that were then combined into 18 (proteome profiling) or six (acetylome profiling) fractions, and dried by vacuum centrifugation.

### Affinity enrichment

For acetylomic analysis, tryptic peptides were dissolved in NETN buffer composed of 1 mM EDTA, 50 mM Tris-HCl, 100 mM NaCl, and 0.5% Nonidet P-40 (pH 8.0) and incubated with pre-washed anti-acetylated lysine antibody beads (PTM Biolabs, Chicago, IL, USA) overnight at 4°C with gentle shaking. The peptides were washed four times in NETN buffer followed by two washes in ddH_2_O. Bound peptides were eluted from the beads with 0.1% trifluoroacetic acid; the eluates were combined and vacuum dried. The eluant was cleaned with C18 ZipTips (Millipore, Billerica, MA, USA) according to the manufacturer's instructions.

### Liquid chromatography–tandem mass spectrometry (LC–MS/MS)

To detect acetylation, the peptide mixture after affinity purification was loaded onto a reversed-phase pre-column (Acclaim PepMap 100; Thermo Fisher Scientific). Peptides were separated using a reversed-phase analytical column (Acclaim PepMap RSLC; Thermo Fisher Scientific). Briefly, the peptide mixture was separated on a linear gradient of 8–25% buffer containing 98% acetonitrile and 0.1% formic acid for 20 min followed by 25–40% buffer for 12 min; the buffer concentration was increased up to 80% in 4 min and held at 80% for the last 4 min. The flow rate was 400 nl/min. Results were analyzed with a Q Exactive Plus hybrid quadrupole-orbitrap mass spectrometer (Thermo Fisher Scientific). Peptides were subjected to nanospray ionization source followed by MS/MS in conjunction with ultra-performance liquid chromatography. Intact peptides were acquired at a resolution of 70,000. Peptides were selected for MS/MS using a normalized collision energy setting of 30, and ion fragments were detected at a resolution of 17,500. A data-dependent “top 20” method was used to identify the most abundant precursor ions (mass range 350–1,800 m/z) above a threshold ion count of 5E3 in the MS survey scan with 15.0-s dynamic exclusion. The electrospray voltage used was 2.0 kV.

MS/MS data were processed using MaxQuant software with an integrated Andromeda search engine (v.1.5.2.8). The tandem MS data were searched against the *C. sinensis* genome dataset (Xia et al., 2017) concatenated with a reverse decoy database. Trypsin/P was specified as the cleavage enzyme allowing up to four missing cleavages, four modifications per peptide, and five charges. The mass error was set to 10 ppm for precursor ions and 0.02 Da for fragment ions. Carbamidomethylation on cysteine was specified as a fixed modification. Oxidation of methionine, acetylation of lysine, and N-terminal acetylation were set as variable modifications. False discovery rate thresholds for peptide, protein, and modification site were specified at 1%. The minimum peptide length was set to 7. All other parameters were set to the default values specified by MaxQuant, and >0.75 was used as the site localization probability. Relative quantification was performed as shown in the Supplementary Materials and Methods, which also describes LC-MS/MS measurements and analysis of data for complete peptide mixtures. Bioinformatics analyses, such as functional annotation and enrichment, motif, and functional interaction network analyses were performed as previously described (Xu et al., 2017).

### Western blotting

Western blotting was performed as previously described (Xu et al., 2017). Anti-acetylated lysine antibody (PTM Biolabs, Chicago, IL, USA; Fang et al., 2015) was used as primary antibody at 1:1,000 dilution. Goat anti-mouse IgG (H+L) (Thermo Fisher Scientific; #31430) was used as the secondary antibody at 1:10,000 dilution.

### Statistical analysis

Pigments and secondary metabolite concentrations were averaged from three independent experiments. Data were analyzed with a two-tailed Student's *t*-test. ^*^ indicates significant difference at *P* < 0.05 and ^**^ indicates significant difference at *P* < 0.01. For TMT quantification, the ratios of the TMT reporter ion intensities in MS/MS spectra (m/z 126 *P* < 0.01. by MaxQuant, and >0.75 was used sion. The electrospray voltage used was 2.0 kV.unique for a given protein were considered for relative quantitation. For each sample, the quantification was normalized using the average ratio of all the unique peptide. Protein quantitation calculated from the median ratio of protein corresponding unique peptides when there were at least two unique peptides in a protein. Two-sample, two-sided *T*-tests were used to compare accumulation of proteins. In general, a significance level of 0.05 was used for statistical testing, and we reported the *P*-value or significance level any time a statistical test was performed. Unless otherwise noted, *P*-values were not adjusted for multiple hypothesis testing.

## Results

### Physiological characterization of ‘Anji Baicha’ developmental stages

**Figure 1:**
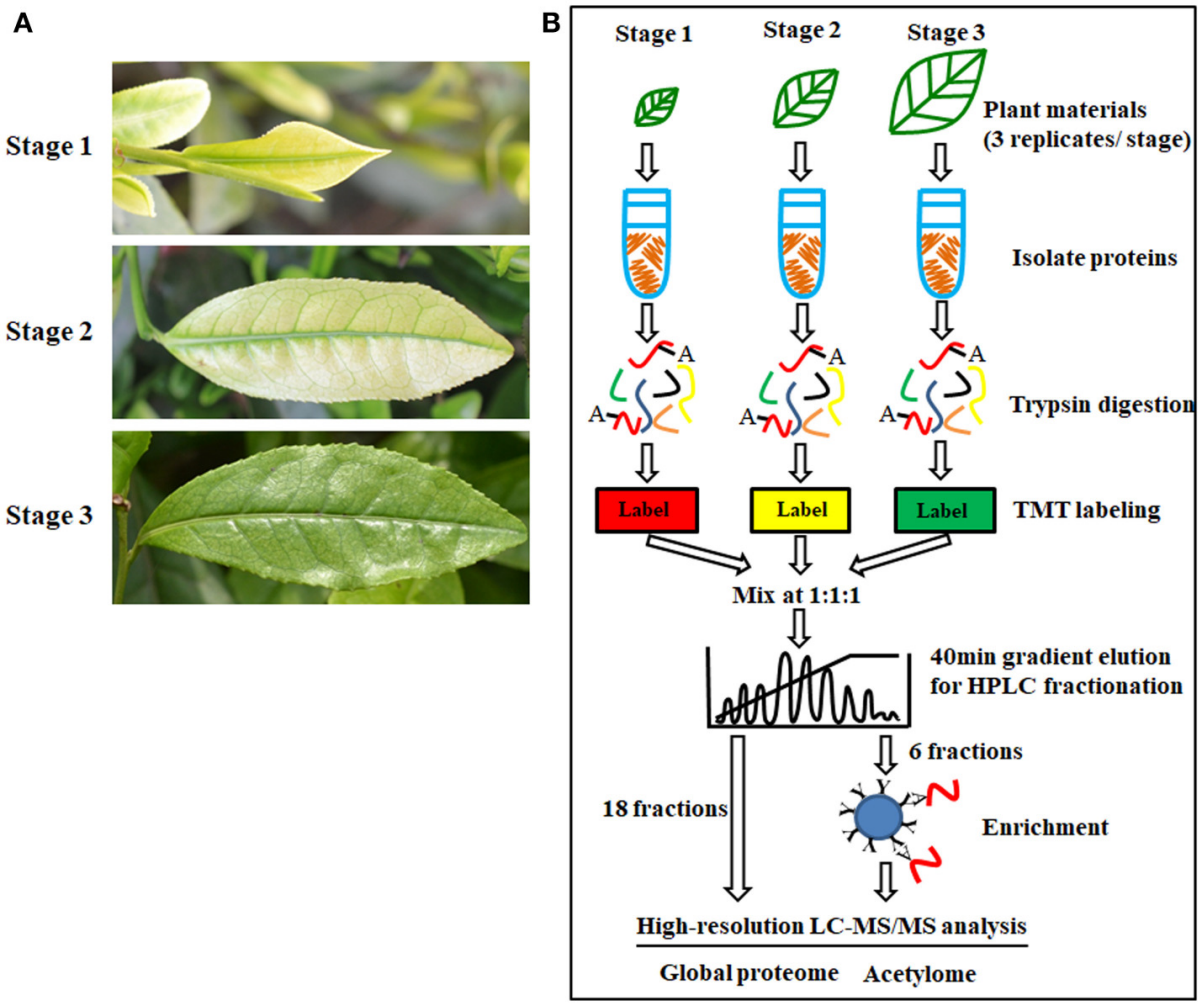
Periodic albinism of ‘Anji Baicha’ leaves and the experimental strategy for the quantification of lysine acetylation and protein accumulation. **(A)** The three developmental stages of ‘Anji Baicha’ leaves. Up to down: pre- albinotic stage (stage 1), albinotic stage (stage 2), re-greening stage (stage 3); **(B)** Experimental strategy for quantifying lysine acetylation and protein accumulation. Proteins were extracted and trypsin digested. The resulting peptides were first separated by HPLC under gradient elution over 40 min to 80 fractions and then were combined into 18 fractions for proteome profiling or 6 fractions for acetylome profiling. Identification and quantification of proteins and modification sites were performed with MaxQuant. False discovery rate (FDR) thresholds for peptides, proteins and modification sites were specified at 1%.

**Figure 2:**
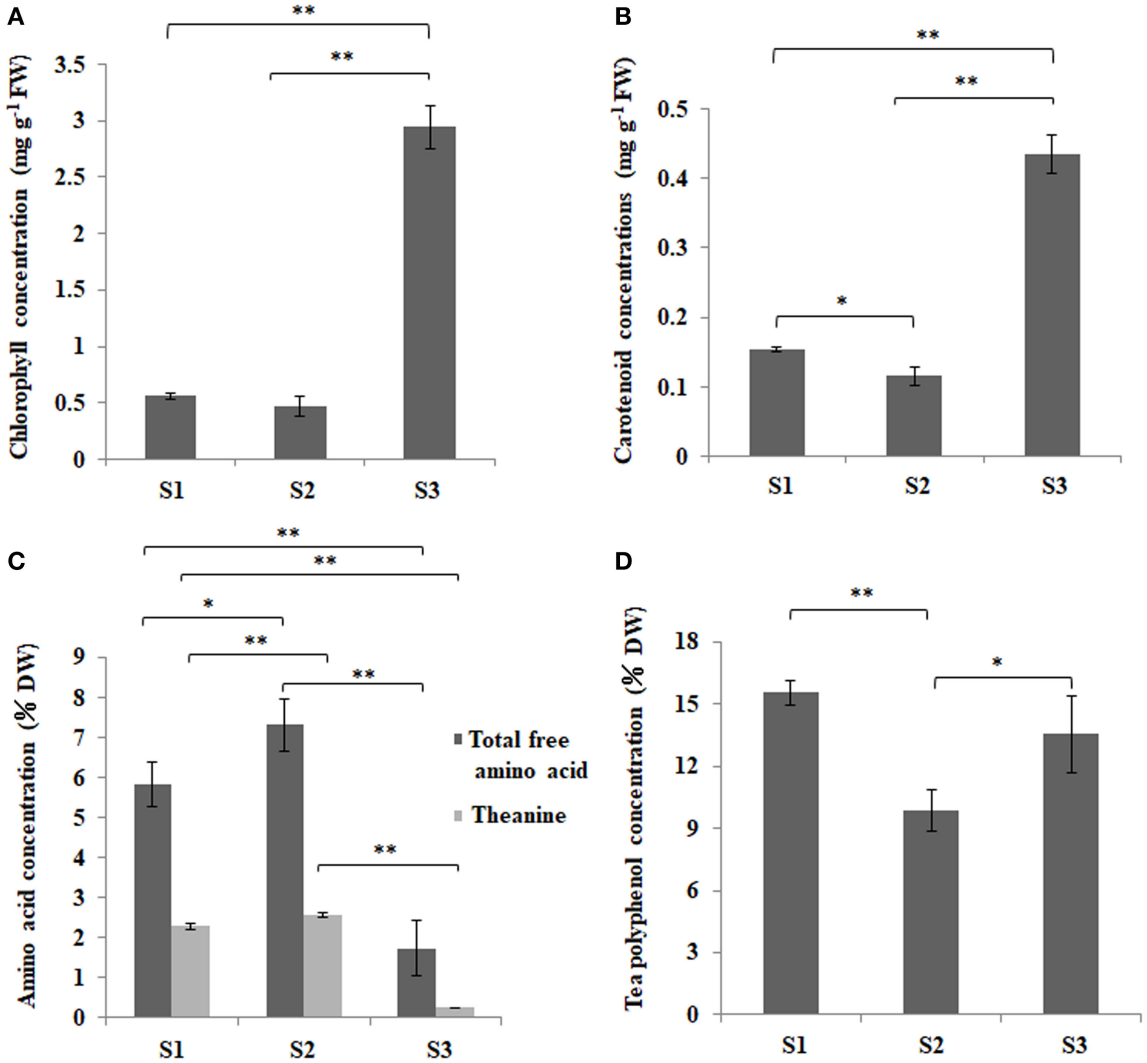
Changes in chlorophyll, carotenoid, free amino acid, and polyphenol concentration in ‘Anji Baicha’ leaves during periodic albinism. **(A)** Chlorophyll; **(B)** carotenoid; **(C)** free amino acid; and **(D)** total tea polyphenol. Data were analyzed from three independent experiments with a two-tailed Student's *t*-test. ^*^Indicates significant difference at *P* < 0.05 and ^**^indicates significant difference at *P* < 0.01.

‘Anji Baicha’ is a green-revertible albino tea cultivar widely grown in China. In this study, we divided ‘Anji Baicha’ leaf development into three stages: stages 1, 2, and 3 represent the pre-albinotic, albinotic, and re-greening stages (Figure 1A). Significant differences in pigment and secondary metabolite concentrations were observed in ‘Anji Baicha’ during periodic albinism (Figure 2). The chlorophyll and carotenoid concentrations were increased (Figures 2A,B), whereas the free amino acid concentration was decreased (Figure 2C) in the re-greening as compared to the other two stages. However, the tea polyphenol concentration was significantly lower in the albinotic stage than in the pre-albinotic and re-greening stages (Figure 2D).

### Proteome profile of each ‘Anji Baicha’ developmental stage

We used high-throughput quantitative proteomics and TMT isobaric labeling technology to identify proteins differentially accumulated in periodic albinism of ‘Anji Baicha’ (Figure 1B). We retained only peptides/proteins detected in at least two replicates, we identified 7,637 proteins and quantified 6,256, of which 3,232 were classified as differentially accumulated proteins (DAPs) with a *P* < 0.05 and fold change >1.5 (Table S1). In total, 1,601 (895 and 706 up- and downregulated), 2,849 (1,122 and 1,727 up- and downregulated, respectively) and 1,347 (495 and 852 up- and downregulated, respectively) unique proteins were differentially accumulated between stage (S)2 vs. S1, S3 vs. S1, and S3 vs. S2, respectively (Figure S1 and Table S1). The volcano plots were shown in Figure S2A.

### Functional classification of DAPs

To clarify the functions of DAPs, we analyzed those showing a >1.5-fold change among the three stages based on Gene Ontology, protein domain, and Kyoto Encyclopedia of Genes Genomes (KEGG) pathway analyses (Figure S3). In the cellular component category, upregulated DAPs were most abundant in external encapsulating structure and extracellular region in S2 vs. S1 as well as in membrane and photosystem in S3 vs. S1 and S3 vs. S2. In contrast, downregulated DAPs were significantly enriched in chromatin and nucleosome, cytoskeleton and eukaryotic translation initiation factor 3 complex, and cell wall and external encapsulating structure in S2 vs. S1, S3 vs. S1, and S3 vs. S2, respectively (Figure S3A). These results suggest that proteins associated with membrane and photosystem may play important roles in the periodic albinism of ‘Anji Baicha.’

In terms of molecular function, upregulated DAPs were most abundant in hydrolase activity in S2 vs. S1, and in oxidoreductase and ATPase activities in S3 vs. S1 and S3 vs. S2, respectively. Downregulated DAPs were most abundant in DNA binding in S2 vs. S1 and in RNA binding and pectinesterase activity in S3 vs. S1 and S3 vs. S2, respectively (Figure S3B). In biological processes, DAPs were enriched in 50 processes, including photosynthesis, flavonoid biosynthesis, peptide metabolism, amide biosynthesis, organic acid catabolism, and others (Figure S3C), indicating that they are involved in a wide range of biological processes. Protein domain analysis showed that upregulated DAPs were highly enriched in the glycoside hydrolase superfamily (2Fe-2S) iron-sulfur domain and heat shock protein 20-like chaperone, whereas downregulated DAPs were most abundant in Mini-chromosome maintenance domain, 50S ribosomal protein L30e-like, and pectin lyase fold in S2 vs. S1, S3 vs. S1, and S3 vs. S2, respectively (Figure S3D).

A KEGG pathway analysis revealed that upregulated DAPs were highly associated with biosynthesis of secondary metabolites and starch and sucrose metabolism in S2 vs. S1, and with photosynthesis (including antenna proteins and carbon fixation in photosynthetic organisms) and carbon metabolism in both S3 vs. S1 and S3 vs. S2. Meanwhile, downregulated DAPs were highly associated with phenylalanine metabolism and flavonoid biosynthesis in both S2 vs. S1 and S3 vs. S1, and with phenylpropanoid biosynthesis and fatty acid elongation in S3 vs. S2 (Figure S3E).

### Analysis of DAPs associated with the photosynthesis and biosynthesis of secondary metabolites

**Figure 3:**
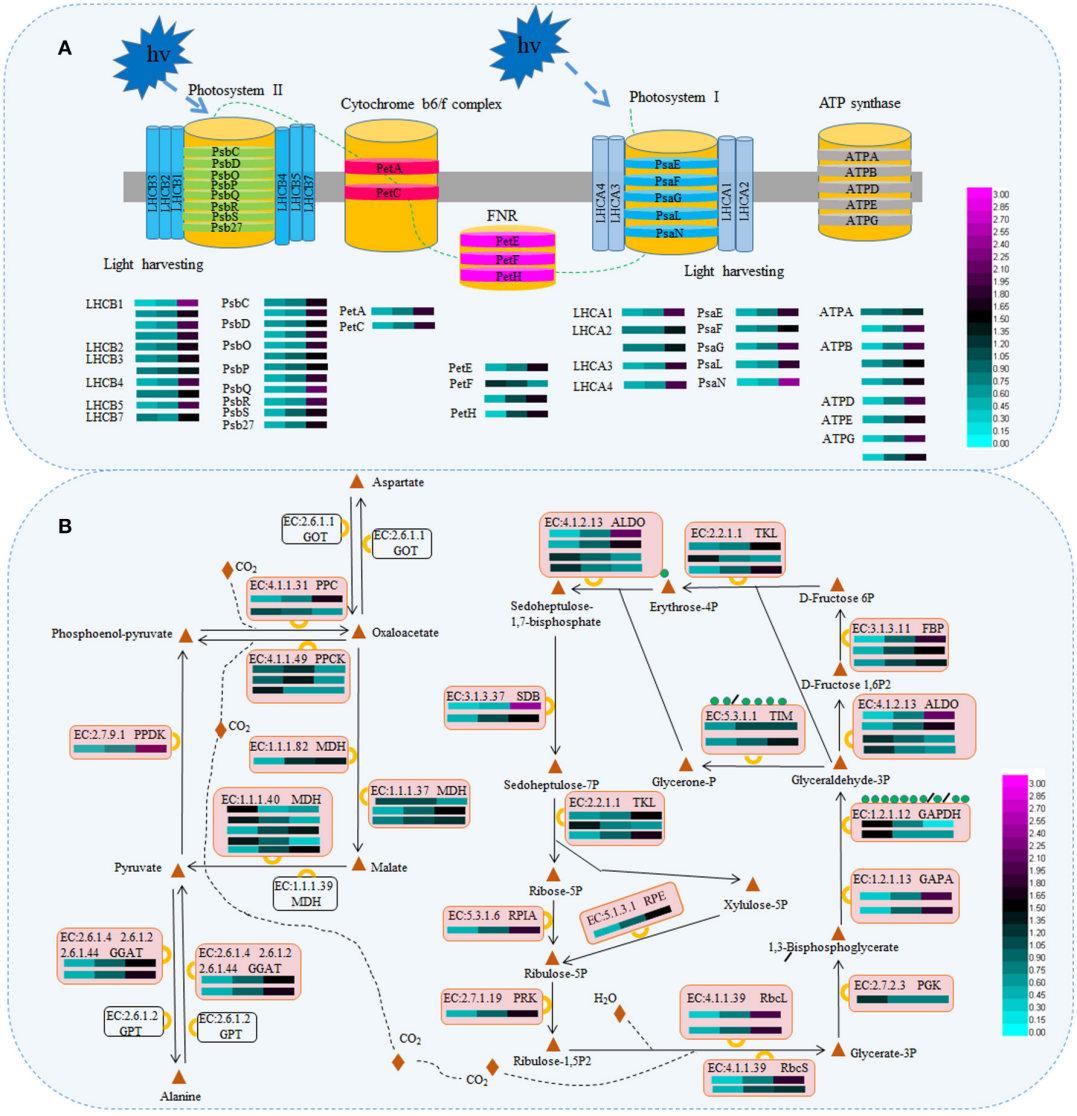
Schematic representation of DAPs associated with photosynthesis **(A)** and carbon fixation **(B)**. The accumulation level of DAPs during three ‘Anji Baicha’ developmental stages is shown using a heatmap (from left to right: Stage 1, Stage 2, Stage 3). PsaE/F/G/L/N, photosystem I subunit IV/III/V/XI/N; PsbO/P/Q, photosystem II oxygen-evolving enhancer protein 1/2/3; Psb C/D/R/S/27, photosystem II CP43/P680 reaction center D2/10 kDa/22 kDa/Psb27 protein; PetA, apocytochrome f; PetC, cytochrome b6-f complex iron-sulfur subunit; PetE, plastocyanin; PetF, ferredoxin; PetH, ferredoxin-NADP+ reductase; ATP A/B/D/E/G, F-type H+-transporting ATPase subunit alpha /beta/ delta/ epsilon/ gamma; LHCA, light-harvesting complex I chlorophyll a/b binding protein; LHCB, light-harvesting complex II chlorophyll a/b binding protein; ALDO, fructose-bisphosphate aldolase; GAPA/GAPDH, glyceraldehyde-3-phosphate dehydrogenase; GGAT, glutamate–glyoxylate aminotransferase; RbcL/S, ribulose-bisphosphate carboxylase large chain/small chain; MDH, malate dehydrogenase; TKL, transketolase; TIM, triosephosphate isomerase; PGK, phosphoglycerate kinase; PRK, phosphoribulokinase; FBP, fructose-1,6-bisphosphatase I; PPC, phosphoenolpyruvate carboxylase; SDB, sedoheptulose-bisphosphatase; GOT, aspartate aminotransferase; GPT, alanine transaminase; PPDK, pyruvate, orthophosphate dikinase; PPCK, phosphoenolpyruvate carboxykinase; RPIA, ribose 5-phosphate isomerase A; RPE, ribulose-phosphate 3-epimerase.

**Figure 4:**
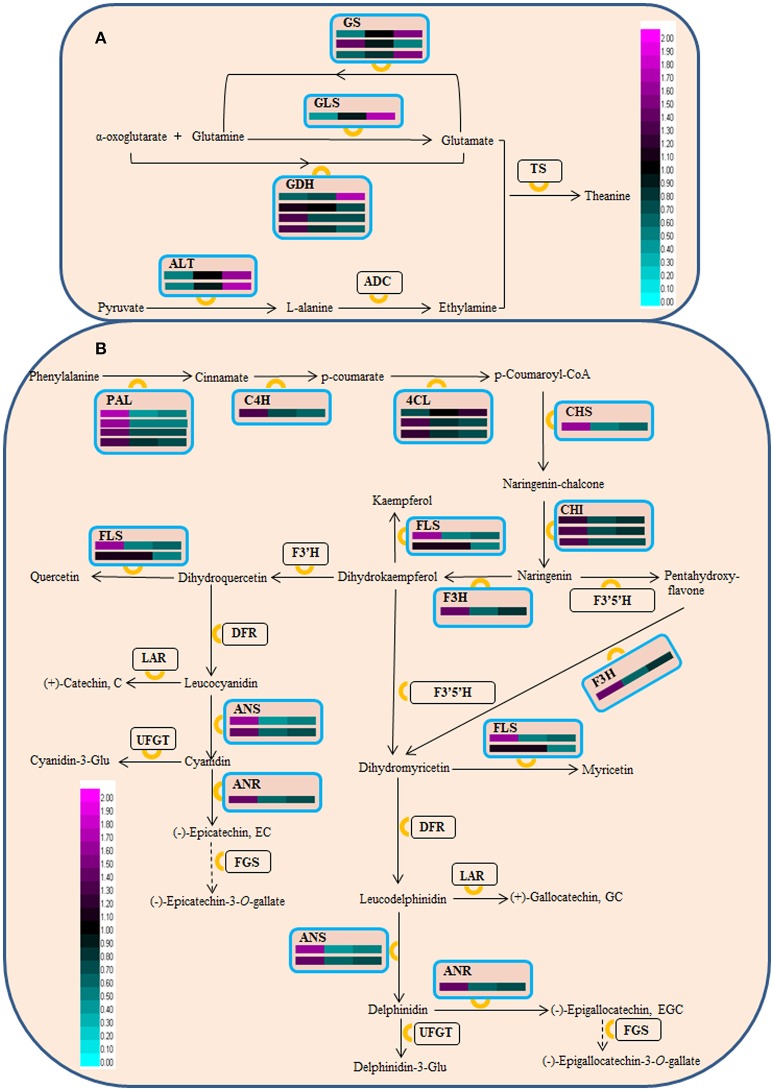
Schematic representation of DAPs associated with theanine **(A)** and flavonoid **(B)** biosynthesis. GS, glutamine synthetase; GLS, glutamate synthase; GDH, glutamate dehydrogenase; ALT, alanine transaminase; ADC, arginine decarboxylase; TS, theanine synthetase; PAL, phenylalanine ammonia-lyase; C4H, cinnamate 4-hydroxylase; 4CL, 4-coumarate coenzyme (Co)A ligase; ANR, anthocyanidin reductase; CHI, chalcone isomerase; CHS, chalcone synthase; DFR, dihydroflavonol 4-reductase; FGS, flavan-3-ol gallate synthase; F3H, flavanone 3-hydroxylase; F3'H, flavonoid 3'-hydroxylase; F3',5'H, flavonoid 3',5'-hydroxylase; FLS, flavonol synthase; LAR, leucocyanidin reductase; ANS, leucoanthocyanidin oxidase; UFGT, UDP-glucose, flavonoid 3-*O*-glucosyl transferase. This pathway was drawn based on Shi et al. (2011) and Li et al. (2015b). The accumulation level of DAPs during three ‘Anji Baicha’ developmental stages is shown using a heatmap (from left to right: Stage 1, Stage 2, Stage 3).

Taking into account the change in pigmentation and secondary metabolite levels during periodic albinism in ‘Anji Baicha’ (Figure 2) and the results of the KEGG pathway analysis (Figure S3E), we examined the accumulation levels of proteins involved in photosynthesis (including antenna proteins and proteins associated with carbon fixation in photosynthetic organisms) and biosynthesis of mostly secondary metabolites in tea plant (Figures 3, 4 and Tables S2, S3). We identified 48 DAPs involved in photosynthesis, including five in photosystem I, 12 in photosystem II, two in the cytochrome b6/f complex, nine that were ATP synthases, 16 in the light-harvesting chlorophyll protein complex, and four that were photosynthetic electron transport proteins (Figure 3 and Table S2). Interestingly, nearly all of these were upregulated in the re-greening stage as compared to the pre-albinotic and albinotic stages. In addition, enzymes involving in the Calvin cycle including malate dehydrogenase (MDH), fructose-1,6-bisphosphatase I (FBP), glutamate–glyoxylate aminotransferase (GGAT), transketolase (TKL), glyceraldehyde 3-phosphate dehydrogenase (GAPDH/GAPA), pyruvate/orthophosphate dikinase (PPDK), phosphoenolpyruvate carboxykinase (PPCK), ribulose-bisphosphate carboxylase small chain and large chain (Rbc), phosphoenolpyruvate carboxylase (PPC), fructose-bisphosphate aldolase (ALDO), sedoheptulose-bisphosphatase (SDB), phosphoglycerate kinase (PGK), phosphoribulokinase (PRK), triosephosphate isomerase (TIM), ribose 5-phosphate isomerase A (RPIA), and ribulose-phosphate 3-epimerase (RPE) were identified as proteins that are differentially accumulated during periodic albinism in ‘Anji Baicha,’ suggesting these enzymes in calvin cycle may play important roles in periodic albinism of ‘Anji Baicha’ (Figure 3 and Table S2).

In this study, we also identified several proteins associated with theanine/flavonoid biosynthesis that were differentially accumulated during periodic albinism in ‘Anji Baicha’ (Figure 4 and Table S3), including four in the theanine biosynthesis pathway—i.e., glutamine synthetase (GS), glutamate synthase (GLS), glutamate dehydrogenase (GDH), and alanine transaminase (ALT). In the flavonoid biosynthesis pathway, 9 enzymes including phenylalanine ammonia-lyase (PAL), cinnamate 4-hydroxylase (C4H), 4-coumarate coenzyme (Co)A ligase (4CL), chalcone synthase (CHS), chalcone isomerase (CHI), flavanone 3-hydroxylase (F3H), flavonol synthase (FLS), anthocyanidin reductase (ANR), and anthocyanidin synthase (ANS) were differentially accumulated.

### Acetylomes of the three leaf development stages of ‘Anji Baicha’

Lysine acetylation regulates protein function in prokaryotes and eukaryotes. However, few acetylated plant proteins have been identified to date, and there are no reports of the acetylome in tea plant. To address this issue, we performed a western blot analysis to identify APs in tea plant and found that many proteins with a wide range of molecular masses were acetylated (Figure S4). By applying TMT quantitative proteomics combined with highly sensitive immune-affinity purification and high-resolution LC-MS/MS (Figure 1B), we identified 3,161 lysine-ASs on 1,752 APs (Table S4).

**Figure 5:**
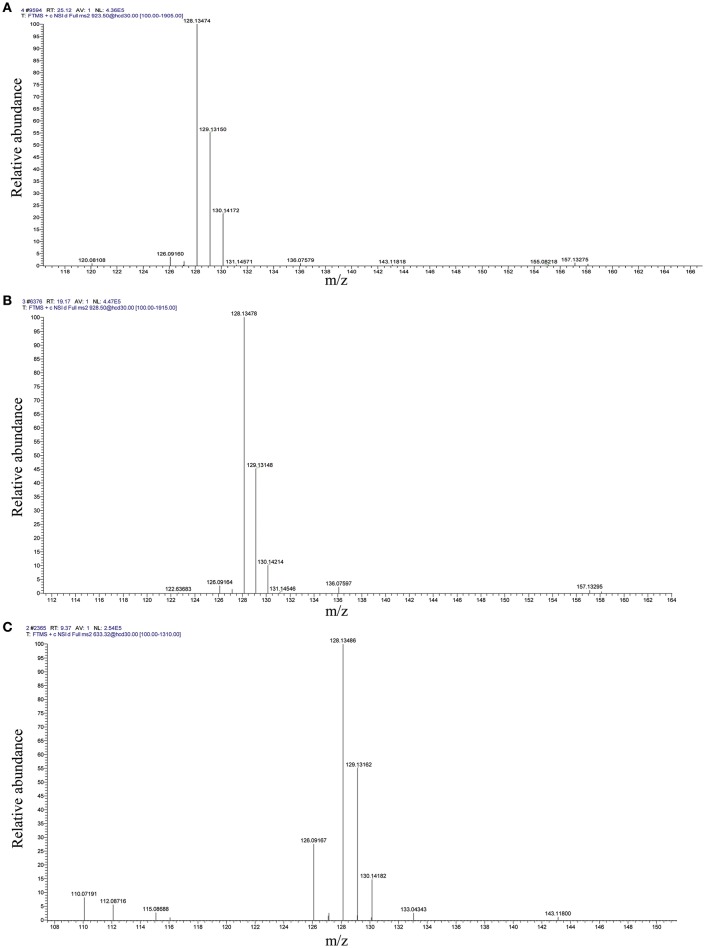
Representative quantitative MS/MS spectra of acetylated peptides with TMT labeling from elongation factor 2 (CSA011928.1). **(A)** K333; **(B)** K361; **(C)** K369. S1:TMT128; S2:TMT129; S3:TMT130.

The distribution of mass error in the proteome and acetylome profiles was approximately zero, and most values were <5 ppm, indicating high accuracy of the MS data (Figure S5). Peptides and acetylated peptides in proteome and acetylome profiles, respectively, showed variable abundance according to their length (Figure S6). The volcano plots of differential ASs were shown in Figure S2B. Quantitative MS/MS spectra of acetylated elongation factor 2 peptides (CSA011928.1) with ASs at K333, K361, and K369 are shown in Figure 5.

### Motif analysis for lysine-acetylated peptides in tea plant

**Figure 6:**
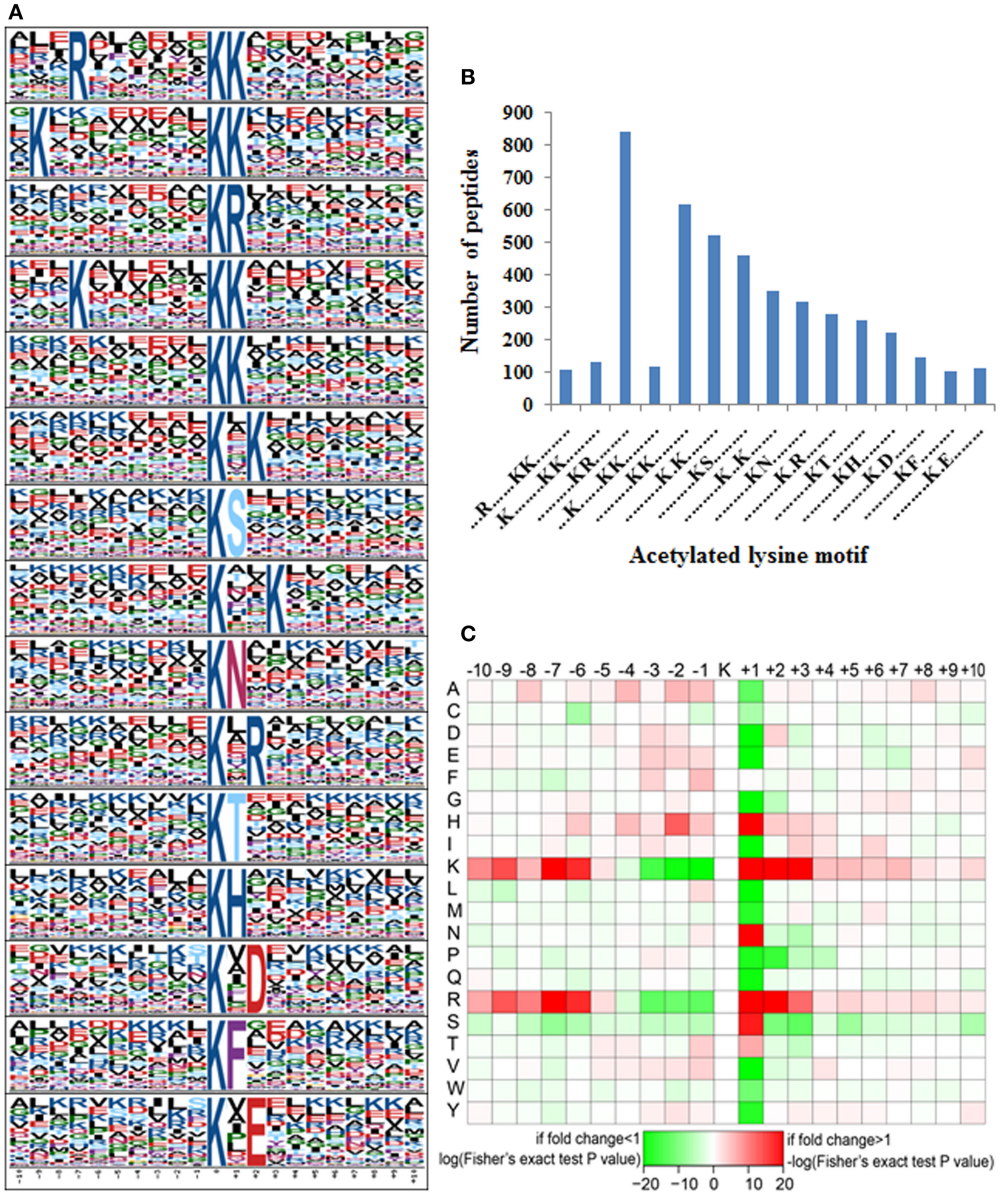
Motif analysis of identified lysine acetylated peptides. **(A)** Probability motifs of tea plant ASs ±10 amino acids residues surrounding the acetylated lysines analyzed by Motif-X. **(B)** Number of identified peptides containing an acetylated lysine in each motif; **(C)** Heat map depicting enrichment (red) or depletion (green) of amino acids around the acetylated lysine.

Accumulating evidence suggests that different species show distinct preferences for amino acid residues at specific positions surrounding acetylated lysines (Kim et al., 2006; Zhang et al., 2009; Okanishi et al., 2013; Pan et al., 2014). Here we performed a search of sequence motifs surrounding all identified acetylated lysines using the Motif-X program and found 15 conserved motifs within the 10 amino acids up- and downstream of these lysines (Figure 6 and Table 1). The sequence logos show a strong preference for lysine and arginine around the acetylated lysines. These amino acid biases may reflect a bona fide preference or may be due to the preference of antibodies used for selective enrichment of acetylated peptides (Xue et al., 2013; Li et al., 2014).

**Table 1:** Motif analysis for the identified lysine-acetylated peptides.

**Motif**	**Motif score**	**Foreground**		**Background**		**Fold increase**
		**Matches**	**Size**	**Matches**	**Size**	
…R……K^ac^K………	24.81	104	4,926	322	69,627	4.57
.K…….K^ac^K………	24.2	127	4,822	459	69,305	3.98
……….K^ac^R………	16	826	4,695	3,860	68,846	3.14
…K……K^ac^K………	22.66	111	3,869	443	64,986	4.21
……….K^ac^K………	16	602	3,758	4,213	64,543	2.45
……….K^ac^.K….…	16	511	3,156	4,411	60,330	2.21
……….K^ac^S………	16	451	2,645	4,417	55,919	2.16
……….K^ac^.K….…	16	338	2,194	3,870	51,502	2.05
……….K^ac^N………	16	305	1,856	2,426	47,632	3.23
……….K^ac^.R….…	16	272	1,551	2,481	45,206	3.2
……….K^ac^T………	16	254	1,279	2,726	42,725	3.11
……….K^ac^H………	16	218	1,025	1,274	39,999	6.68
……….K^ac^.D….…	16	139	807	2,199	38,725	3.03
……….K^ac^F………	16	103	668	1,976	36,526	2.85
……….K^ac^.E….…	16	113	565	2,940	34,550	2.35

*K^ac^ represents the acetylated lysine and “.” represents any amino acid*.

### KEGG pathway analysis of APs with differentially ASs

Among the 3,161 identified lysine-ASs on 1,752 APs, 2,869 ASs on 1,612 APs were quantifiable (Table S4). We found that normalized acetylation levels of many ASs were altered across ‘Anji Baicha’ developmental stages. At 468 ASs, acetylation levels were altered by >1.5-fold (Figure S7 and Table S4), with 88 and 38 up- and downregulated, respectively, in S2 vs. S1; 242 and 167 up- and downregulated, respectively, in S3 vs. S1; and 123 and 115 up- and downregulated, respectively, in S3 vs. S2 (Table S4). Interestingly, more ASs were significantly altered in S3 vs. S2 and S3 vs. S1 as compared to S2 vs. S1, consistent with the extent of leaf albinism at each stage (Figure 2A). Moreover, 35 ASs were changed among S2 vs. S1, S3 vs. S1, and S3 vs. S2, indicating an association between the three comparisons and the progression of a biological process (Figure S7).

**Figure 7:**
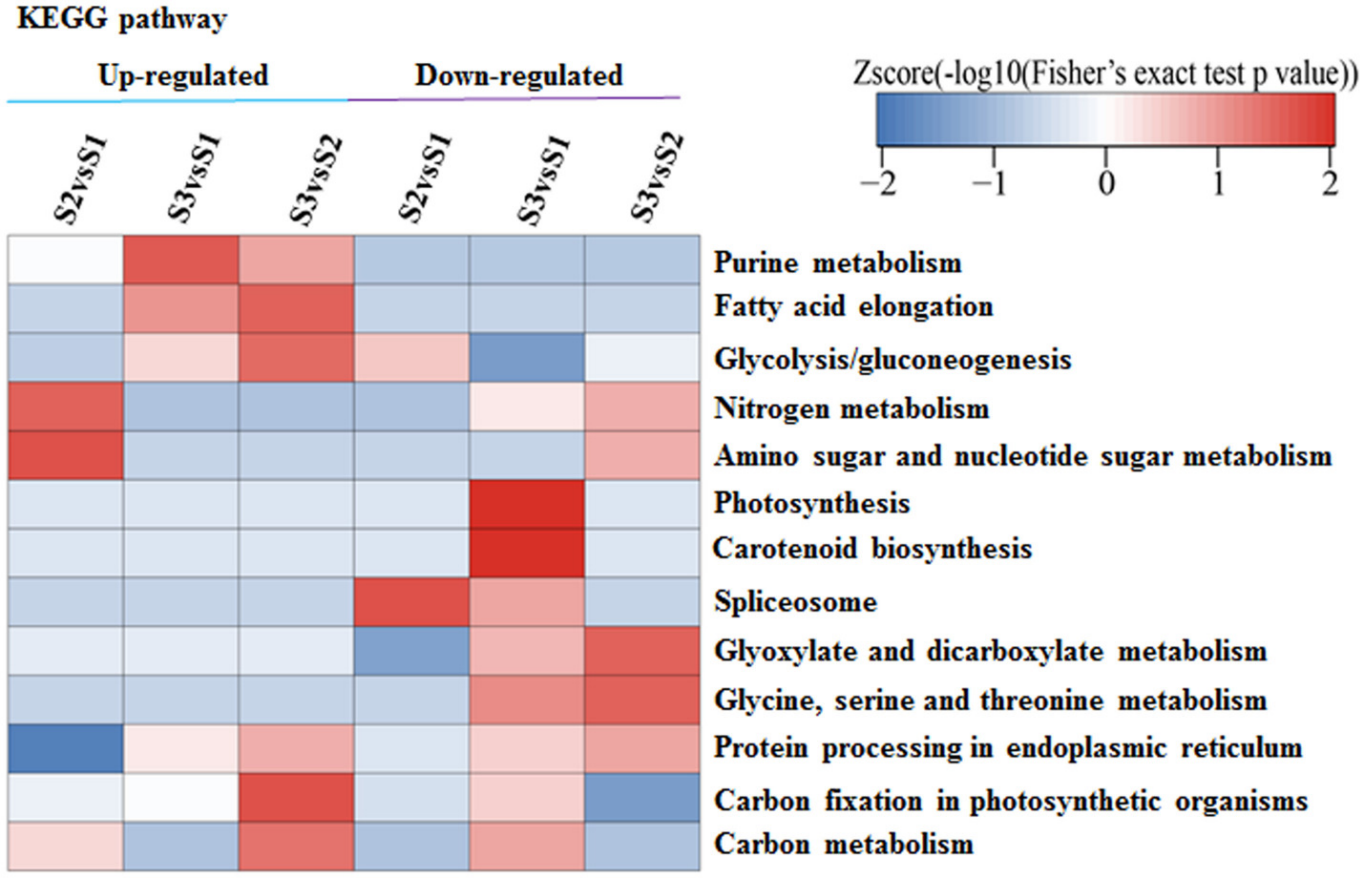
KEGG analysis of APs with altered acetylation levels. The acetylation levels of ASs were normalized by protein accumulated levels. The *p*-values were transformed into z-scores prior to hierarchical clustering analysis.

A KEGG pathway analysis revealed that APs with increased or decreased acetylation levels were closely associated with photosynthesis, carbon fixation in photosynthetic organisms, carotenoid biosynthesis, purine metabolism, and fatty acid elongation (Figure 7). These results provide evidence that acetylation is related to periodic albinism in ‘Anji Baicha.’ ASs and APs associated with photosynthesis and some secondary metabolites biosynthesis were shown in Table S5 and Table 6.

### Overlap between succinylome and acetylome among three ‘Anji Baicha’ developmental stages

**Figure 8:**
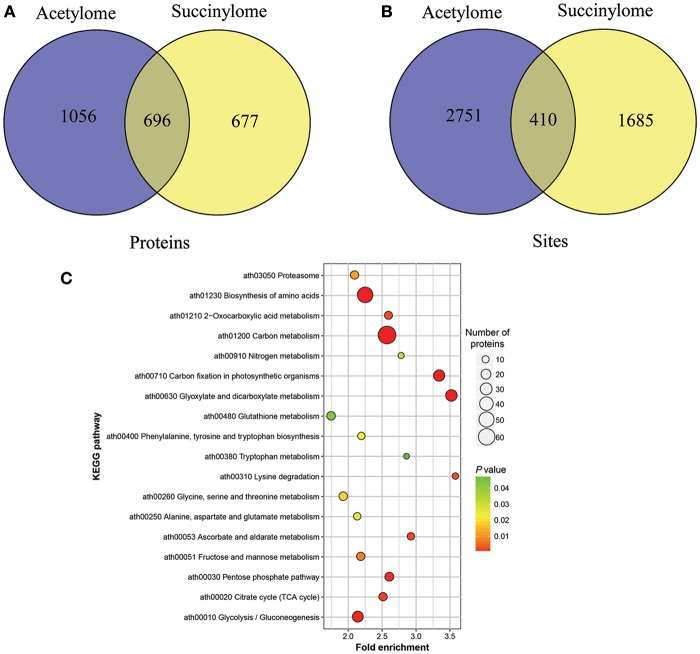
Overlap in succinylome and acetylome of ‘Anji Baicha.’ **(A)** Venn diagram showing the comparison of proteins in succinylome and acetylome of ‘Anji Baicha’; **(B)** Venn diagram showing the comparison of sites in succinylome and acetylome of ‘Anji Baicha’; **(C)** KEGG analysis of the proteins identified with both ASs and SSs. The acetylation or succinylation levels of ASs or SSs were normalized by protein accumulated levels. The *p*-values were used to hierarchical clustering analysis.

**Figure 9:**
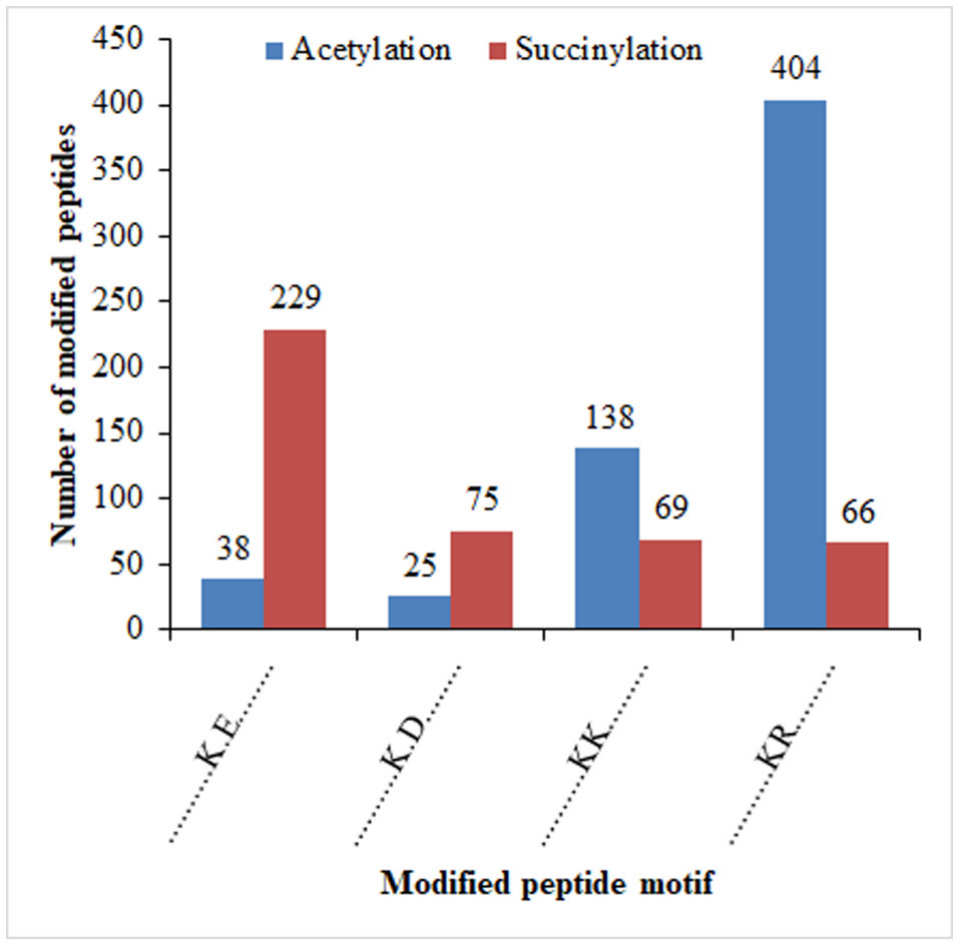
Significant motifs for overlapped peptides identified by Motif-X software.

It was reported that lysine succinylation extensively overlaps with acetylation in prokaryotes and eukaryotes (Weinert et al., 2013). To further confirm the relationship of succinylaion and acetylation in ‘Anji Baicha,’ we compared the ASs identified in this study to our previously identified succinylation sites (SSs) (Xu et al., 2017). To keep consistency, we re-analyzed the SSs against the tea genome dataset (Xia et al., 2017) using MaxQuant. We found that 696 proteins and 410 sites were both succinylated and acetylated in ‘Anji Baicha’ (Figures 8A,B and Tables S7, S8), providing further support to the previous studies (Weinert et al., 2013). KEGG pathway analysis revealed that overlapped proteins were mostly found to be involved in carbon metabolism, biosynthesis of amino acids and carbon fixation proteins in photosynthetic organisms (Figure 8C). Motif analysis showed that four motifs (……….K.E…….,……….K.D…….,……….KK………, and……….KR………) were overlapped between succinylome and acetylome (Figure 9).

## Discussion

This study analyzed the protein and acetylome profiles of ‘Anji Baicha’ during periodic albinism. Proteins that were differentially accumulated in the three ‘Anji Baicha’ leaf development stages were mostly found to be involved in photosynthesis (including antenna and carbon fixation proteins in photosynthetic organisms), biosynthesis of secondary metabolites, and carbon metabolism, among others. A large number of ASs and APs were identified in tea plant for the first time. Many APs related to photosynthesis, carotenoid biosynthesis, and purine metabolism showed altered acetylation levels during periodic albinism in ‘Anji Baicha.’ Additionally, overlap between succinylome and acetylome among three ‘Anji Baicha’ developmental stages were found.

A previous study using two-dimensional electrophoresis identified 26 DAPs implicated in ‘Anji Baicha’ periodic albinism (Li et al., 2011). However, this method is not suitable for analyzing proteins with low abundance. In contrast, TMT quantitative proteomics has many advantages, including higher accuracy and coverage and sensitivity for proteins accumulated at low levels. Using this approach, we identified 3,232 proteins that are differentially accumulated during ‘Anji Baicha’ periodic albinism, including most of those reported by the earlier study (Li et al., 2011).

Acetylation has attracted widespread attention of researchers in recent years. In *Salmonella enterica*, 235 ASs on 191 APs have been identified (Wang et al., 2010). In addition, 226 ASs on 137 APs (Liu et al., 2014) and 1,128 ASs on 658 APs (Xie et al., 2015) have been reported in *Mycobacterium tuberculosis* H37Ra and H37Rv, respectively. A total of 776 ASs on 513 APs are present in *Synechocystis* sp. PCC 6,803 (Mo et al., 2015), and 3,600 ASs on 1,750 APs are known to exist in human cells (Choudhary et al., 2009). In *Streptomyces roseosporus* and *Bacillus amyloliquefaciens*, 1,143 and 3,268 lysine ASs were identified on 667 and 1,254 APs, respectively (Liao et al., 2014; Mo et al., 2015; Liu et al., 2016). In contrast, fewer APs have been identified in plants—i.e., 64 ASs on 57 APs (Wu et al., 2011), 91 ASs on 74 APs (Finkemeier et al., 2011), 243 ASs on 120 APs (König et al., 2014) and 2,152 ASs on 1,022 APs (Hartl et al., 2017) in *Arabidopsis*; 60 ASs on 44 APs in rice (Nallamilli et al., 2014); 400 ASs on 245 APs in soybean (Smith-Hammond et al., 2014b), 664 ASs on 358 APs in pea (Smith-Hammond et al., 2014a); 138 ASs in grape (Melo-Braga et al., 2012); and 1,392 ASs on 684 APs in strawberry (Fang et al., 2015). Here we identified 3,161 lysine ASs on 1,752 APs, which is more than what has been previously reported in plants. Possible reasons for the variable abundance of ASs and APs among plant species are the different experimental conditions, including the tissue sampled and setting. For example, the grape data were from the fruit; *Arabidopsis* data were from young seedling or mature leaves or isolated mitochondria; soybean and pea data were from immature seeds and purified mitochondria, respectively; and data from strawberry as well as those from the tea plant in our study were from the leaves. Thus, none of the results reported thus far reflect the overall acetylome. However, our findings represent the first lysine acetylome map of tea plants.

Pigmentation in most plant species is determined by the relative chlorophyll, carotenoid, and flavonoid/anthocyanin contents. Several studies have suggested that leaf color is associated with chloroplast development, pigment metabolism, and signal transduction (Wettstein et al., 1995; Sundberg et al., 1997; Beale, 1999; Terry and Kendrick, 1999; Kumar and Soll, 2000; Ishikawa et al., 2001; Eckhardt et al., 2004; Zhou et al., 2017). As a periodic albino tea cultivar, the chlorophyll and carotenoid concentrations of ‘Anji Baicha’ were much higher in the re-greening than in pre-albinotic and albinotic stages (Figures 2A,B); thus, the lower chlorophyll concentration and altered chlorophyll/carotenoid ratio may be responsible for the albino phenotype. A KEGG pathway analysis of APs showed significant enrichment in carotenoid biosynthesis pathway components, suggesting that they are related to the albino phenotype in ‘Anji Baicha.’ Carotenoids absorb and convert light energy into chemical energy in photosynthesis. Rice plants deficient in genes encoding carotenoid biosynthesis pathway enzymes exhibited an albino phenotype; a decreased carotenoid content in these mutants resulted in reduced chlorophyll content due to photooxidative decomposition (Fang et al., 2008). This maybe can explain the decreased chlorophyll content in the albinotic stage of ‘Anji Baicha.’ In this study, we identified 13 DAPs in the carotenoid biosynthesis pathway (Table S9). One of these—zeaxanthin epoxidase (Zep)—plays an important role in carotenoid composition. The hydroxylated β rings of zeaxanthin are subject to sequential epoxidation by Zep to yield antheraxanthin followed by violaxanthin. Interestingly, the protein accumulation level of Zep was increased by 10-fold during re-greening stage as compared to the pre-albinotic stage in ‘Anji Baicha’ (Table S9), indicating that it could modulate periodic albinism in this cultivar. Carotenoid isomerase (CrtISO) is essential for the biosynthesis of carotenoid precursors of abscisic acid (ABA). Rice OsCRTISO mutants exhibited an albino phenotype (Fang et al., 2008); however, we did not observe the change in CrtISO protein accumulation level across the three leaf development stages of ‘Anji Baicha,’ although CrtISO acetylation level differed between the re-greening and albinotic stages (Tables S6, S9), suggesting that changes in CrtISO acetylation level might involve in periodic albinism in ‘Anji Baicha.’

Photosynthesis is a crucial biological process in higher plants, as plants harvest energy derived from the sun depending on it. Many chlorophyll-deficient mutants are unable to survive resulted from destructive photosynthesis (Jung et al., 2003; Gothandam et al., 2005; Lee et al., 2005). Photosynthesis involves four major protein complexes: photosystem I, photosystem II, the cytochrome b6/f complex, and ATP synthase. In this study, we identified five DAPs in photosystem I, 12 in photosystem II, two in the cytochrome b6/f complex, and nine ATP synthases. Interestingly, all of these were found to be upregulated in the re-greening stage of ‘Anji Baicha’ relative to the other stages (Figure 3 and Table S2), suggesting that photosynthesis was increased in accordance with the increased chlorophyll concentration in this stage (Figure 2A). Leaf color is affected by temperature in many plant species; changes in temperature lead to temporary leaf color variations in chlorophyll-deficient mutants, including ‘Anji Baicha’ (Cheng et al., 1999; Li, 2002; Li et al., 2011). Thus, given the increased chlorophyll concentration in the re-greening stage, ‘Anji Baicha’ may exhibit enhanced photosynthesis via upregulation of related proteins in order to survive, in contrast to most chlorophyll-deficient mutants.

The light-harvesting chlorophyll protein complex and photosynthetic electron transport proteins also play important roles in photosynthesis. In this study, we identified 16 DAPs in the light-harvesting chlorophyll protein complex and four in photosynthetic electron transport proteins (Figure 3 and Table S2). Upregulated DAPs in S3 vs. S2 and S3 vs. S1 were enriched in antenna proteins (Figure S3), suggesting a link to the periodic albino phenotype. Light-harvesting complexes (LHCs) are major constituents of the antenna systems of higher plants. In ‘Anji Baicha,’ all 16 differentially accumulated LHC proteins were found markedly upregulated in the re-greening stage as compared to the pre-albinotic and albinotic stages (Figure 3 and Table S2), suggesting an enhanced capacity for chlorophyll capture. Additionally, several ASs have been identified in LHCs in wheat, including LHCA1, LHCB3, LHCB5, and LHCB6 (Zhang et al., 2016), while LHCB1 and LHCB5 in *Arabidopsis* contained lysine ASs (Wu et al., 2011). In this study, we identified several ASs in LHCA1, LHCA3, and LHCB1–5 of tea plant (Table S5). These results highlight the widespread nature of LHC acetylation in plants. Interestingly, LHCA1 acetylation levels (CSA024064.1, K184) were higher in the re-greening than in the pre-albinotic and albinotic stages in ‘Anji Baicha,’ suggesting that LHC acetylation is associated with the periodic albino phenotype.

Calvin cycle in photosynthesis converts carbon dioxide into organic compounds. In this study, a number of enzymes involving in Calvin cycle—including MDH, FBP, GGAT, TKL, GAPDH/GAPA, PPDK, PPCK, Rbc, PPC, ALDO, SDB, PGK, PRK, TIM, RPIA, and RPE—were identified as DAPs in tea plant. There is accumulating evidence linking SDB to plant productivity. For example, in an *Arabidopsis SDB* loss-of-function mutant, reactive oxygen species-induced oxidative damage affected growth and development via inhibition of carbon assimilation (Liu et al., 2012). In our study, SDB accumulation was higher during re-greening than in the other stages of leaf development. In the case of CSA028009.1, accumulation was 7- and 4-fold higher in the re-greening as compared to the pre-albinotic and albinotic stages, respectively (Table S2). The opposite trend was observed for GAPDH (CSA016761.1; 5- and 3-fold lower, respectively) (Table S2), suggesting that the two proteins are differentially regulated in ‘Anji Baicha’ during periodic albinism.

Metabolic enzymes can be modified by lysine acetylation, thus affecting enzymatic activity or stability (Chen et al., 2010; Zhao et al., 2010; Finkemeier et al., 2011; Gao et al., 2016). For example, the activity of Rbc—a rate-limiting enzyme in the Calvin cycle—was shown to be inhibited by acetylation (Finkemeier et al., 2011; Gao et al., 2016). In this study, a number of enzymes involved in the Calvin cycle were identified as APs in tea plant (Table S5). Similar results have been reported in cyanobacterium, *Arabidopsis*, and wheat (Wu et al., 2011; Mo et al., 2015; Zhang et al., 2016). We also found that acetylation levels of FBP (CSA030111.1, K25) were altered in the albinotic stage, while re-greening affected ALDO (CSA005116.1, K103), GAPDH (CSA016761.1, K93), and TIM (CSA003118.1, K261) acetylation, suggesting that these enzymes coordinately modulate periodic albinism in ‘Anji Baicha.’

Numerous genes related to secondary metabolite pathways have been identified in the tea plant (Shi et al., 2011; Tai et al., 2015). Theanine is an abundant, non-protein-derived free amino acid in tea plants. Tea flavonoids comprise a broad collection of tea polyphenol secondary metabolites, including anthocyanidins, flavanols, flavanones, isoflavones, flavonols, and flavones (Lin et al., 1996; Shi et al., 2011); these compounds contribute to tea quality. In ‘Anji Baicha,’ the total free amino acid and theanine concentrations were decreased in the re-greening as compared to the pre-albinotic and albinotic stages (Figure 2C), whereas tea polyphenol concentration was lower in the albinotic stage than in the other two stage (Figure 2D), consistent with previous reports (Li et al., 1996; Du et al., 2006; Xiong et al., 2013). Thus, the rich amino acid content and modest tea polyphenol content in the albinotic stage may underlie the higher quality of ‘Anji Baicha’ when new shoots become albino.

Theanine biosynthesis involves arginine decarboxylase, ALT, GLS, GS, GDH, and theanine synthetase (Li et al., 2015b). Here we identified four key enzymes (GLS, GS, GDH, and ALT) that were differentially accumulated during periodic albinism in ‘Anji Baicha.’ GS catalyzes the conversion of glutamate to glutamine. In our previous transcriptome analysis, GS abundance was found to be increased in the re-greening as compared to the pre-albinotic and albinotic stages (Li et al., 2016). In this study, GS protein level varied, but no significant change in acetylation level was observed (Table S3, S4). These results suggest that dynamic alterations in the RNA and protein accumulation of GS contribute to differences in theanine abundance across the three stages of ‘Anji Baicha’ leaf development.

We identified 18 DAPs corresponding to 9 enzymes in the flavonoid biosynthesis pathway (Figure 4 and Table S3), and 46 ASs in the flavonoid biosynthesis pathway (Table S6). Among these ASs, 14 mapped to PAL, one to C4H, and three to 4CL, which are located upstream of this pathway and promote the conversion of phenylalanine to p-coumaryol-CoA. Seven ASs mapped to CHS, which catalyzes the production of chalcone from p-coumaryol-CoA; and two mapped to CHI, which catalyzes the conversion of naringenin-chalcone to naringenin. Additionally, F3H, FLS, ANS, and ANR had several lysine ASs. These results suggest that acetylation is linked to flavonoid biosynthesis. Of the 46 lysine ASs identified in this pathway, six were significantly altered during periodic albinism in ‘Anji Baicha.’ The acetylation level of PAL (CSA016076.1, K40, K540) and 4CL (CSA003473.1, K512) was decreased during re-greening, whereas that of CHS (CSA CSA024718.1, K62) and F3H (CSA004930.1, K110, K336) was decreased in the albino (Table S6). Thus, acetylation of PAL, 4CL, CHS, and F3H may contribute to the differences in flavonoid abundance across the three ‘Anji Baicha’ developmental stages. It is worth noting that all of the identified ASs with altered acetylation levels are upstream of the flavonoid biosynthesis pathway except for ANR and ANS, suggesting that this PTM is more important at the initial steps of this process. However, leaf color ultimately results from interactions between numerous genetic, developmental, and environmental factors. Therefore, additional experiments are needed to clarify the mechanistic basis for periodic albinism in ‘Anji Baicha.’

It was reported that lysine succinylation extensively overlaps with acetylation in prokaryotes and eukaryotes (Weinert et al., 2013). In this work, 696 proteins were detected being both succinylated and acetylated, with 410 overlapped sites (Figures 8A,B and Tables S7, S8), providing further support to the previous studies (Weinert et al., 2013). These results suggested that crosstalk between succinylation and acetylation might exist in ‘Anji Baicha.’ These overlapped proteins were mostly found to be involved in carbon metabolism, biosynthesis of amino acids and carbon fixation proteins in photosynthetic organisms (Figure 8C), suggesting that the SSs and ASs might cooperate or compete with each other on the same proteins in these pathways. Additionally, we found that most of the overlapped proteins encode metabolic enzymes, which highlighted the regulation role of PTMs in central metabolic pathway. Motif analysis showed that four motifs (……….K.E…….,……….K.D…….,……….KK………, and……….KR………) were overlapped between succinylome and acetylome (Figure 9), suggesting that the overlaps were preferentially to occur on lysine and arginine at position +1, and on glutamic acid and aspartic acid at position +2 around the modified lysines. These results suggested that the lysine located at the upstream of the polar acidic/basic amino acid were more easily to be modified by both acetylation and succinylation, which is in accord with the previous report (He et al., 2016).

## Conclusion

In summary, the results presented here provide the first comprehensive analysis of the proteome and acetylome during different leaf development stages in the tea plant. A large number of DAPs and APs with increased/decreased acetylation were found to be associated with photosynthesis and secondary metabolite biosynthesis, suggesting that the accumulation or acetylation level of these proteins modulate periodic albinism in ‘Anji Baicha.’ Additionally, overlap between succinylome and acetylome among three ‘Anji Baicha’ developmental stages were found. Our study provides novel insight into the mechanisms of albescence in this cultivar that could benefit future breeding strategies for generating tea plants with different leaf colors or improved leaf quality.

## Author contributions

Y-XX and LC conceived of and designed the experiment; Y-XX, WC, C-LM, S-YS, Y-YZ, and L-QZ performed the experiments; Y-XX, Y-YZ and L-QZ analyzed the data; and Y-XX and LC wrote the paper.

### Conflict of interest statement

The authors declare that the research was conducted in the absence of any commercial or financial relationships that could be construed as a potential conflict of interest.

## Supplementary material

Figure S1 Number of DAPs among three stages of “Anji Baicha” leaf development.

Figure S2 The volcano plots of differential DAPs **(A)** and ASs **(B)**.

Figure S3 Enrichment-based clustering analysis of DAPs. **(A)** Cellular component; **(B)** molecular function; **(C)** biological process; **(D)** protein domain; and **(E)** KEGG pathway.

Figure S4 Western blot analysis of acetylation levels at three stages of ‘Anji Baicha’ leaf development.

Figure S5 Mass error distribution of peptides identified in the proteome **(A)** and acetylome **(B)** profiles (based on three biological replicates).

Figure S6 Distribution of peptides in proteome profiles **(A–C)** and acetylated peptides in acetylome profiles **(D–F)** according to length (based on three biological replicates).

Figure S7 Comparative analysis of differentially ASs among the three ‘Anji Baicha’ developmental stages.

Table S1 Proteomic profiles at three stages of ‘Anji Baicha’ leaf development.

Table S2 DAPs in photosynthesis and carbon fixation pathways.

Table S3 DAPs in theanine and flavonoid biosynthetic pathways.

Table S4 Identified lysine-acetylated proteins at three stages of ‘Anji Baicha’ leaf development.

Table S5 ASs and APs in photosynthesis and carbon fixation pathways.

Table S6 ASs and APs in theanine, flavonoid, and carotenoid biosynthetic pathways.

Table S7 Overlapped proteins in succinylome and acetylome of ‘Anji Baicha.’

Table S8 Overlapped sites in succinylome and acetylome of ‘Anji Baicha.’

Table S9 DAPs in carotenoid biosynthetic pathways.

## References

[B1] Beale S. I. (1999). Enzymes of chlorophyll biosynthesis. Photosyn. Res. 6, 43–73. 10.1023/A:1006297731456

[B2] Benhamed M. Bertrand C. Servet C. Zhou D. X. (2006). Arabidopsis GCN5, HD1, and TAF1/HAF2 interact to regulate histone acetylation required for light-responsive gene expression. Plant Cell 18, 2893–2903. 10.1105/tpc.106.043489 17085686 PMC1693931

[B3] Chen J. Wang P. Mi H. L. Chen G. Y. Xu D. Q. (2010). Reversible association of ribulose-1, 5-bisphosphate carboxylase/oxygenase activase with the thylakoid membrane depends upon the ATP level and pH in rice without heat stress. J. Exp. Bot. 61, 2939–2950. 10.1093/jxb/erq122 20478969 PMC2892142

[B4] Chen L. Zhou Z. X. Yang Y. J. (2007). Genetic improvement and breeding of tea plant (*Camellia sinensis*) in China: from individual selection to hybridization and molecular breeding. Euphytica 154, 239–248. 10.1007/s10681-006-9292-3

[B5] Cheng H. Li S. F. Chen M. Yu F. L. Ya J. Liu Y. M. (1999). Physiological and biochemical essence of the extraordinary characters of Anji Baicha. J. Tea Sci. 19, 87–92.

[B6] Choudhary C. Kumar C. Gnad F. Nielsen M. L. Rehman M. Walthe T. C.. (2009). Lysine acetylation targets protein complexes and co-regulates major cellular functions. Science 325, 834–840. 10.1126/science.1175371 19608861

[B7] Deng W. Liu C. Pei Y. Deng X. Niu L. Cao X. (2007). Involvement of the histone acetyltransferase AtHAC1 in the regulation of flowering time via repression of FLOWERING LOCUS C in Arabidopsis. Plant Physiol. 143, 1660–1668. 10.1104/pp.107.095521 17416640 PMC1851829

[B8] Du Y. Y. Liang Y. R. Wang H. Wang K. R. Lu J. L. Zhang G. H. (2006). A study on the chemical composition of albino tea cultivars. J. Hortic. Sci. Biotechnol. 81, 809–812. 10.1080/14620316.2006.11512142

[B9] Eckhardt U. Grimm B. Hortensteiner S. (2004). Recent advances in chlorophyll biosynthesis and breakdown in higher plants. Plant Mol. Biol. 56, 1–14. 10.1007/s11103-004-2331-3 15604725

[B10] Fang J. Chai C. L. Qian Q. Li C. Tang J. Sun L.. (2008). Mutations of genes in synthesis of the carotenoid precursors of ABA lead to pre-harvest sprouting and photo-oxidation in rice. Plant J. 54, 177–189. 10.1111/j.1365-313X.2008.03411.x 18208525 PMC2327239

[B11] Fang X. Chen W. Zhao Y. Ruan S. Zhang H. Yan C.. (2015). Global analysis of lysine acetylation in strawberry leaves. Front. Plant Sci. 6:739. 10.3389/fpls.2015.00739 26442052 PMC4569977

[B12] Finkemeier I. Laxa M. Miguet L. Howden A. J. Sweetlove L. J. (2011). Proteins of diverse function and subcellular location are lysine acetylated in *Arabidopsis*. Plant Physiol. 155, 1779–1790. 10.1104/pp.110.171595 21311031 PMC3091095

[B13] Gao X. Hong H. Li W. C. Yang L. Huang J. Xiao Y. L.. (2016). Downregulation of rubisco activity by non-enzymatic acetylation of RbcL. Mol. Plant 9, 1018–1027. 10.1016/j.molp.2016.03.012 27109602

[B14] Gothandam K. M. Kim E. S. Cho H. Chung Y. Y. (2005). OsPPR1, a pentatricopeptide repeat protein of rice is essential for the chloroplast biogenesis. Plant Mol. Biol. 7, 421–433. 10.1007/s11103-005-5702-5 16021404

[B15] Hartl M. Füßl M. Boersema P. J. Jost J. O. Kramer K. Bakirbas A.. (2017). Lysine acetylome profiling uncovers novel histone deacetylase substrate proteins in Arabidopsis. Mol. Syst. Biol. 13:949. 10.15252/msb.20177819 29061669 PMC5658702

[B16] He D. Wang Q. Li M. Damaris R. N. Yi X. Cheng Z.. (2016). Global proteome analyses of lysine acetylation and succinylation reveal the widespread involvement of both modification in metabolism in the embryo of germinating rice seed. J. Proteome Res. 15, 879–890. 10.1021/acs.jproteome.5b00805 26767346

[B17] Hu Y. Qin F. Huang L. Sun Q. Li C. (2009). Rice histone deacetylase genes display specific expression patterns and developmental functions. Biochem. Biophys. Res. Commun. 388, 266–271. 10.1016/j.bbrc.2009.07.162 19664599

[B18] Ishikawa A. Okamoto H. Iwasaki Y. Asahi T. (2001). A deficiency of coproporphyrinogen III oxidase causes lesion formation in *Arabidopsis*. Plant J. 27, 89–99. 10.1046/j.1365-313x.2001.01058.x 11489187

[B19] Jung K. H. Hur J. Ryu C. H. Choi Y. Chung Y. Y. Miyao A.. (2003). Characterization of a rice chlorophyll-deficient mutant using the T-DNA gene-trap system. Plant Cell Physiol. 44, 63–72. 10.1093/pcp/pcg064 12773632

[B20] Kim S. C. Sprung R. Chen Y. Xu Y. Ball H. Pei J.. (2006). Substrate and functional diversity of lysine acetylation revealed by a proteomics survey. Mol. Cell 23, 607–618. 10.1016/j.molcel.2006.06.026 16916647

[B21] Komiya R. Ikegami A. Tamaki S. Yokoi S. Shimamoto K. (2008). Hd3a and RFT1 are essential for flowering in rice. Development 135, 767–774. 10.1242/dev.008631 18223202

[B22] König A. C. Hartl M. Boersema P. J. Mann M. Finkemeier I. (2014). The mitochondrial lysine acetylome of *Arabidopsis*. Mitochondrion 19(Pt B), 252-60. 10.1016/j.mito.2014.03.004 24727099

[B23] Kouzarides T. (2000). Acetylation: a regulatory modification to rival phosphorylation? EMBO J. 19, 1176–1179. 10.1093/emboj/19.6.1176 10716917 PMC305658

[B24] Kumar A. M. Söll D. (2000). Antisense HEMA1 RNA expression inhibits heme and chlorophyll biosynthesis in *Arabidopsis*. Plant Physiol. 122, 49–55. 10.1104/pp.122.1.49 10631248 PMC58843

[B25] Lee K. K. Workman J. L. (2007). Histone acetyltransferase complexes: one size doesn't fit all. Nat. Rev. Mol. Cell Biol. 8, 284–295. 10.1038/nrm2145 17380162

[B26] Lee S. Kim J. H. Yoo E. S. Lee C. H. Hirochika H. An G. (2005). Differential regulation of chlorophyll a oxygenase genes in rice. Plant Mol. Biol. 9, 805–818. 10.1007/s11103-005-2066-9 15952067

[B27] Li C. F. Xu Y. X. Ma J. Q. Jin J. Q. Huang D. J. Yao M. Z.. (2016). Biochemical and transcriptomic analyses reveal different metabolite biosynthesis profiles among three color and developmental stages in ‘Anji Baicha’ (*Camellia sinensis*). BMC Plant Biol. 16:195. 10.1186/s12870-016-0885-2 27609021 PMC5015330

[B28] Li C. F. Yao M. Z. Ma C. L. Ma J. Q. Jin J. Q. Chen L. (2015a). Differential metabolic profiles during the albescent stages of ‘Anji Baicha’ (*Camellia sinensis*). PLoS ONE 10:e0139996. 10.1371/journal.pone.0139996 26444680 PMC4622044

[B29] Li C. F. Zhu Y. Yu Y. Zhao Q. Y. Wang S. J. Wang X. C.. (2015b). Global transcriptome and gene regulation network for secondary metabolite biosynthesis of tea plant (*Camellia sinensis*). BMC Genomics 16:560. 10.1186/s12864-015-1773-0 26220550 PMC4518527

[B30] Li Q. Huang J. Liu S. Li J. Yang X. Liu Y.. (2011). Proteomic analysis of young leaves at three developmental stages in an albino tea cultivar. Proteome Sci. 9:44. 10.1186/1477-5956-9-44 21806834 PMC3162873

[B31] Li S. F. (2002). Studies on the mechanism of the leaf color change in Anjibaicha (*Camellia sinensis*). J. China Inst. Metrol 13, 214–217.

[B32] Li S. F. Cheng H. Yu F. L. (1996). The change of amino acid in the stage albinism of White leaf No.1. J. Tea Sci. 16, 153–154.

[B33] Li X. Hu X. Wan Y. Xie G. Li X. Chen D.. (2014). Systematic identification of the lysine succinylation in the protozoan parasite *Toxoplasma gondii*. J. Proteome Res. 13, 6087–6095. 10.1021/pr500992r 25377623

[B34] Liao G. Xie L. Li X. Cheng Z. Xie J. (2014). Unexpected extensive lysine acetylation in the trump-card antibiotic producer *Streptomyces roseosporus* revealed by proteome-wide profiling. J. Proteomics 25, 260–269. 10.1016/j.jprot.2014.04.017 24768905

[B35] Lichtenthaler H. Wellburn A. (1983). Determinations of total carotenoids and chlorophylls a and b of leaf extracts in different solvents. Analysis (Peach) 11, 591–592. 10.1042/bst0110591

[B36] Lin Y. L. Juan I. M. Chen Y. L. Liang Y. C. Lin J. K. (1996). Composition of polyphenols in fresh tea leaves and associations of their oxygen-radical-absorbing capacity with antiproliferative actions in fibroblast cells. J. Agric. Food Chem. 44, 1387–1394. 10.1021/jf950652k

[B37] Liu F. Yang M. Wang X. Yang S. Gu J. Zhou J.. (2014). Acetylome analysis reveals diverse functions of lysine acetylation in *Mycobacterium tuberculosis*. Mol. Cell. Proteomics 13, 3352–3366. 10.1074/mcp.M114.041962 25180227 PMC4256489

[B38] Liu L. Wang G. Song L. Lv B. Liang W. (2016). Acetylome analysis reveals the involvement of lysine acetylation in biosynthesis of antibiotics in *Bacillus amyloliquefaciens*. Sci. Rep. 6:20108. 10.1038/srep20108 26822828 PMC4731788

[B39] Liu X. L. Yu H. D. Guan Y. Li J. K. Guo F. Q. (2012). Carbonylation and loss-of-function analyses of SBPase reveal its metabolic interface role in oxidative stress, carbon assimilation, and multiple aspects of growth and development in *Arabidopsis*. Mol. Plant 5, 1082–1099. 10.1093/mp/sss012 22402261

[B40] Ma C. L. Chen L. Wang X. C. Jin J. Q. Ma J. Q. Yao M. Z. (2012). Differential expression analysis of different albescent stages of ‘Anji Baicha’ (*Camellia sinensis* (L.) O. *Kuntze)* using cDNA microarray. Sci. Hortic. 148, 246–254. 10.1016/j.scienta.2012.09.033

[B41] Melo-Braga M. N. Verano-Braga T. León I. R. Antonacci D. Nogueira F. C. Thelen J. J.. (2012). Modulation of protein phosphorylation, N-glycosylation and Lys acetylation in grape (*Vitis vinifera*) mesocarp and exocarp owing to Lobesia botrana infection. Mol. Cell. Proteomics 11, 945–956. 10.1074/mcp.M112.020214 22778145 PMC3494143

[B42] Mo R. Yang M. Chen Z. Cheng Z. Yi X. Li C.. (2015). Acetylome analysis reveals the involvement of lysine acetylation in photosynthesis and carbon metabolism in the model cyanobacterium *Synechocystis* sp. PCC 6803. J. Proteome Res. 14, 1275–1286. 10.1021/pr501275a 25621733

[B43] Nallamilli B. R. Edelmann M. J. Zhong X. Tan F. Mujahid H. Zhang J.. (2014). Global analysis of lysine acetylation suggests the involvement of protein acetylation in diverse biological processes in rice (*Oryza sativa*). PLoS ONE 9:e89283. 10.1371/journal.pone.0089283 24586658 PMC3930695

[B44] Okanishi H. Kim K. Masui R. Kuramitsu S. (2013). Acetylome with structural mapping reveals the significance of lysine acetylation in *Thermus thermophilus*. J. Proteome Res. 12, 3952–3968. 10.1021/pr400245k 23901841

[B45] Pan J. Ye Z. Cheng Z. Peng X. Wen L. Zhao F. (2014). Systematic analysis of the lysine acetylome in *Vibrio parahemolyticus*. J. Proteome Res. 13, 3294–3302. 10.1021/pr500133t 24874924

[B46] Pandey R. Müller A. Napoli C. A. Selinger D. A. Pikaard C. S. Richards E. J.. (2002). Analysis of histone acetyltransferase and histone deacetylase families of *Arabidopsis thaliana* suggests functional diversification of chromatin modification among multicellular eukaryotes. Nucleic Acids Res. 30, 5036–5055. 10.1093/nar/gkf660 12466527 PMC137973

[B47] Prabakaran S. Lippens G. Steen H. Gunawardena J. (2012). Post-translational modification: nature's escape from genetic imprisonment and the basis for dynamic information encoding. Wiley Interdiscip. Rev. Syst. Biol. Med. 4, 565–583. 10.1002/wsbm.1185 22899623 PMC3473174

[B48] Semane B. Dupae J. Cuypers A. Noben J. P. Tuomainen M. Tervahauta A.. (2010). Leaf proteome responses of *Arabidopsis thaliana* exposed to mild cadmium stress. J. Plant Physiol. 167, 247–254. 10.1016/j.jplph.2009.09.015 20005002

[B49] Servet C. Conde e Silva N. Zhou D. X. (2010). Histone acetyltransferase AtGCN5/HAG1 is a versatile regulator of developmental and inducible gene expression in *Arabidopsis*. Mol. Plant 3, 670–677. 10.1093/mp/ssq018 20457643

[B50] Shahbazian M. D. Grunstein M. (2007). Functions of site-specific histone acetylation and deacetylation. Annu. Rev. Biochem. 76, 75–100. 10.1146/annurev.biochem.76.052705.162114 17362198

[B51] Shi C. Y. Yang H. Wei C. L.. (2011). Deep sequencing of the *Camellia sinensis* transcriptome revealed candidate genes for major metabolic pathways of tea-specific compounds. BMC Genomics 12:131. 10.1186/1471-2164-12-131 21356090 PMC3056800

[B52] Smith-Hammond C. L. Hoyos E. Miernyk J. A. (2014a). The pea seedling mitochondrial Nε-lysine acetylome. Mitochondrion 19(Pt B), 154–65. 10.1016/j.mito.2014.04.012 24780491

[B53] Smith-Hammond C. L. Swatek K. N. Johnston M. L. Thelen J. J. Miernyk J. A. (2014b). Initial description of the developing soybean seed protein Lys-Nε-acetylome. J. Proteomics 96, 56–66. 10.1016/j.jprot.2013.10.038 24211405

[B54] Sundberg E. Slagter J. G. Fridborg I. Cleary S. P. Robinson C. Coupland G. (1997). *Albino3*, an *Arabidopsis* nuclear gene essential for chloroplast differentiation, encodes a chloroplast protein that shows homology to proteins present in bacterial membranes and yeast mitochondria. Plant Cell 9, 717–730. 10.2307/3870427 9165749 PMC156951

[B55] Tai Y. Wei C. Yang H. Zhang L. Chen Q. Deng W.. (2015). Transcriptomic and phytochemical analysis of the biosynthesis of characteristic constituents in tea (*Camellia sinensis*) compared with oil tea (*Camellia oleifera*). BMC Plant Biol. 15:190. 10.1186/s12870-015-0574-6 26245644 PMC4527363

[B56] Tan F. Zhang K. Mujahid H. Verma D. P. Peng Z. (2011). Differential histone modification and protein expression associated with cell wall removal and regeneration in rice (*Oryza sativa*). J. Proteome Res. 10, 551–563. 10.1021/pr100748e 20958091

[B57] Tan H. P. Xu W. P. Zhao A. P. Sun D. F. Tan F. Y. (2014). Determination of Free Amino Acids in Plants. Beijing: China Standards Press, GB/T 30987–2014.

[B58] Terry M. J. Kendrick R. E. (1999). Feedback inhibition of chlorophyll synthesis in the phytochrome chromophore-deficient aurea and yellow-green-2 mutants of tomato. Plant Physiol. 119, 143–152. 10.1104/pp.119.1.143 9880355 PMC32213

[B59] Tsuji H. Saika H. Tsutsumi N. Hirai A. Nakazono M. (2006). Dynamic and reversible changes in histone H3-Lys4 methylation and H3 acetylation occurring at submergence-inducible genes in rice. Plant Cell Physiol. 47, 995–1003. 10.1093/pcp/pcj072 16774928

[B60] Wang Q. Zhang Y. Yang C. Xiong H. Lin Y. Yao J.. (2010). Acetylation of metabolic enzymes coordinates carbon source utilization and metabolic flux. Science 327, 1004–1007. 10.1126/science.1179687 20167787 PMC4183141

[B61] Weinert B. T. Schölz C. Wagner S. A. Iesmantavicius V. Su D. Daniel J. A.. (2013). Lysine succinylation is a frequently occurring modification in prokaryotes and eukaryotes and extensively overlaps with acetylation. Cell Rep. 4, 842–851. 10.1016/j.celrep.2013.07.024 23954790

[B62] Wettstein D. V. Gough S. Kannangara C. G. (1995). Chlorophyll Biosynthesis. Plant Cell 7, 1039–1057. 10.1105/tpc.7.7.1039 12242396 PMC160907

[B63] Wu X. Oh M. H. Schwarz E. M. Larue C. T. Sivaguru M. Imai B. S.. (2011). Lysine acetylation is a widespread protein modification for diverse proteins in *Arabidopsis*. Plant Physiol. 155, 1769–1778. 10.1104/pp.110.165852 21311030 PMC3091122

[B64] Xia E. H. Zhang H. B. Sheng J. Li K. Zhang Q. J. Kim C.. (2017). The tea tree genome provides insights into tea flavor and independent evolution of caffeine biosynthesis. Mol. Plant 10, 866–877. 10.1016/j.molp.2017.04.002 28473262

[B65] Xie L. Wang X. Zeng J. Zhou M. Duan X. Li Q.. (2015). Proteome-wide lysine acetylation profiling of the human pathogen *Mycobacterium tuberculosis*. Int. J. Biochem. Cell Biol. 59, 193–202. 10.1016/j.biocel.2014.11.010 25456444

[B66] Xiong L. Li J. Li Y. Yuan L. Liu S. Huang J. A.. (2013). Dynamic changes in catechin levels and catechin biosynthesis-related gene expression in albino tea plants (*Camellia sinensis* L.). Plant Physiol. Biochem. 71, 132–143. 10.1016/j.plaphy.2013.06.019 23911731

[B67] Xiong Y. Peng X. Cheng Z. Liu W. Wang G. L. (2016). A comprehensive catalog of the lysine-acetylation targets in rice (*Oryza sativa*) based on proteomic analyses. J. Proteomics 138, 20–29. 10.1016/j.jprot.2016.01.019 26836501

[B68] Xu Y. X. Shen C. J. Ma J. Q. Chen W. Mao J. Zhou Y. Y.. (2017). Quantitative succinyl-proteome profiling of *Camellia sinensis* cv. ‘Anji Baicha’ during periodic albinism. Sci. Rep. 7:1873. 10.1038/s41598-017-02128-x 28500349 PMC5431936

[B69] Xue B. Jeffers V. Sullivan W. J. Uversky V. N. (2013). Protein intrinsic disorder in the acetylome of intracellular and extracellular *Toxoplasma gondii*. Mol. Biosyst. 9, 645–657. 10.1039/c3mb25517d 23403842 PMC3594623

[B70] Yang X. J. (2004). Lysine acetylation and the bromodomain: a new partnership for signaling. Bioessays 26, 1076–1087. 10.1002/bies.20104 15382140

[B71] Yuan L. Xiong L. G. Deng T. T. Wu Y. Li J. Liu S. Q.. (2015). Comparative profiling of gene expression in *Camellia sinensis* L. *cultivar* AnJiBaiCha leaves during periodic albinism. Gene 561, 23–29. 10.1016/j.gene.2015.01.007 25576956

[B72] Zhang J. Sprung R. Pei J. Tan X. Kim S. Zhu H.. (2009). Lysine acetylation is a highly abundant and evolutionarily conserved modification in *Escherichia coli*. Mol. Cell. Proteomics 8, 215–225. 10.1074/mcp.M800187-MCP200 18723842 PMC2634580

[B73] Zhang Y. Song L. Liang W. Mu P. Wang S. Lin Q. (2016). Comprehensive profiling of lysine acetylproteome analysis reveals diverse functions of lysine acetylation in common wheat. Sci. Rep. 6:21069. 10.1038/srep21069 26875666 PMC4753473

[B74] Zhao S. Xu W. Jiang W. Yu W. Lin Y. Zhang T.. (2010). Regulation of cellular metabolism by protein lysine acetylation. Science 327, 1000–1004. 10.1126/science.1179689 20167786 PMC3232675

[B75] Zhou D. X. Hu Y. F. (2010). Regulatory function of histone modification in controlling rice gene expression and plant growth. Rice 3, 103–111. 10.1007/s12284-010-9045-8

[B76] Zhou W. L. Xu J. F. Xu L. (2008). Determination of Total Polyphenols and Catechins Content in Tea. Beijing: China Standards Press, GB/T. 8313–2008.

[B77] Zhou Q. Chen Z. Lee J. Li X. Sun W. (2017). Proteomic analysis of tea plants (*Camellia sinensis*) with purple young shoots during leaf development. PLoS ONE 12:e0177816. 10.1371/journal.pone.0177816 28520776 PMC5433784

