# Corrigendum: Proteome and Acetyl-Proteome Profiling of *Camellia sinensis* cv. ‘Anji Baicha’ during Periodic Albinism Reveals Alterations in Photosynthetic and Secondary Metabolite Biosynthetic Pathways

**DOI:** 10.3389/fpls.2018.00147

**Published:** 2018-02-07

**Authors:** Yan-Xia Xu, Wei Chen, Chun-Lei Ma, Si-Yan Shen, Yan-Yan Zhou, Lian-Qi Zhou, Liang Chen

**Affiliations:** ^1^Key Laboratory of Tea Biology and Resources Utilization, Ministry of Agriculture, National Center for Tea Improvement, Tea Research Institute of the Chinese Academy of Agricultural Sciences, Hangzhou, China; ^2^Jingjie PTM Biolab (Hangzhou) Co., Ltd., Hangzhou, China

**Keywords:** acetylation, acetylome, albino, metabolism, PTM, proteomic, tea, overlap

In the original article there was a typographic error with the name of the tea cultivar used in our study.

We incorrectly used “Anjin Baicha” throughout the manuscript; the correct spelling is “Anji Baicha.”

The authors apologize for this error and state that this does not change the scientific conclusions of the article in any way.

The original article has been updated.

## Conflict of interest statement

The authors declare that the research was conducted in the absence of any commercial or financial relationships that could be construed as a potential conflict of interest.

