# Supplementary material for: Proteome and Acetyl-Proteome Profiling of Camellia sinensis cv. ‘Anji Baicha’ during Periodic Albinism Reveals Alterations in Photosynthetic and Secondary Metabolite Biosynthetic Pathways
Source: Front Plant Sci. 2017 Dec 11;8:2104. doi: 10.3389/fpls.2017.02104 (PMC5732353; doi:10.3389/fpls.2017.02104)
Supplement: Figure S1 — Number of DAPs among three stages of “Anji Baicha” leaf development. [file DataSheet1.PDF]

Supplementary Material

Proteome and Acetyl-proteome Profiling of *Camellia sinensis* cv.'Anji Baicha' During Periodic Albinism Reveals Alterations in Photosynthetic and Secondary Metabolite Biosynthetic Pathways

Yan-Xia Xu[1+], Wei Chen[1+], Chun-Lei Ma[1+], Si-Yan Shen[1], Yan-Yan Zhou[2], Lian-Qi Zhou[2], Liang Chen[1]*

1. National Center for Tea Improvement, Tea Research Institute of the Chinese Academy of Agricultural Sciences/ Key Laboratory of Tea Biology and Resources Utilization, Ministry of Agriculture, 9 South Meiling Road, Hangzhou 310008, China
2. Jingjie PTM Biolab (Hangzhou) Co., Ltd., Hangzhou 310018, China

Corresponding Author：

[*] Liang Chen, E-mail:liangchen@tricaas.com; Tel:+86-571-8665 2835, Fax:+86-571-8665 0056

+These authors contributed equally.

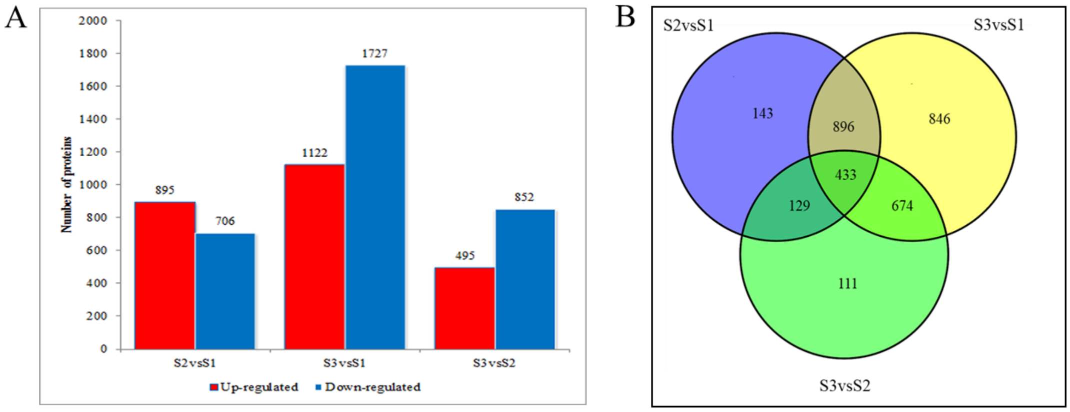

Figure S1. Number of DAPs among three stages of 'Anji Baicha' leaf development.

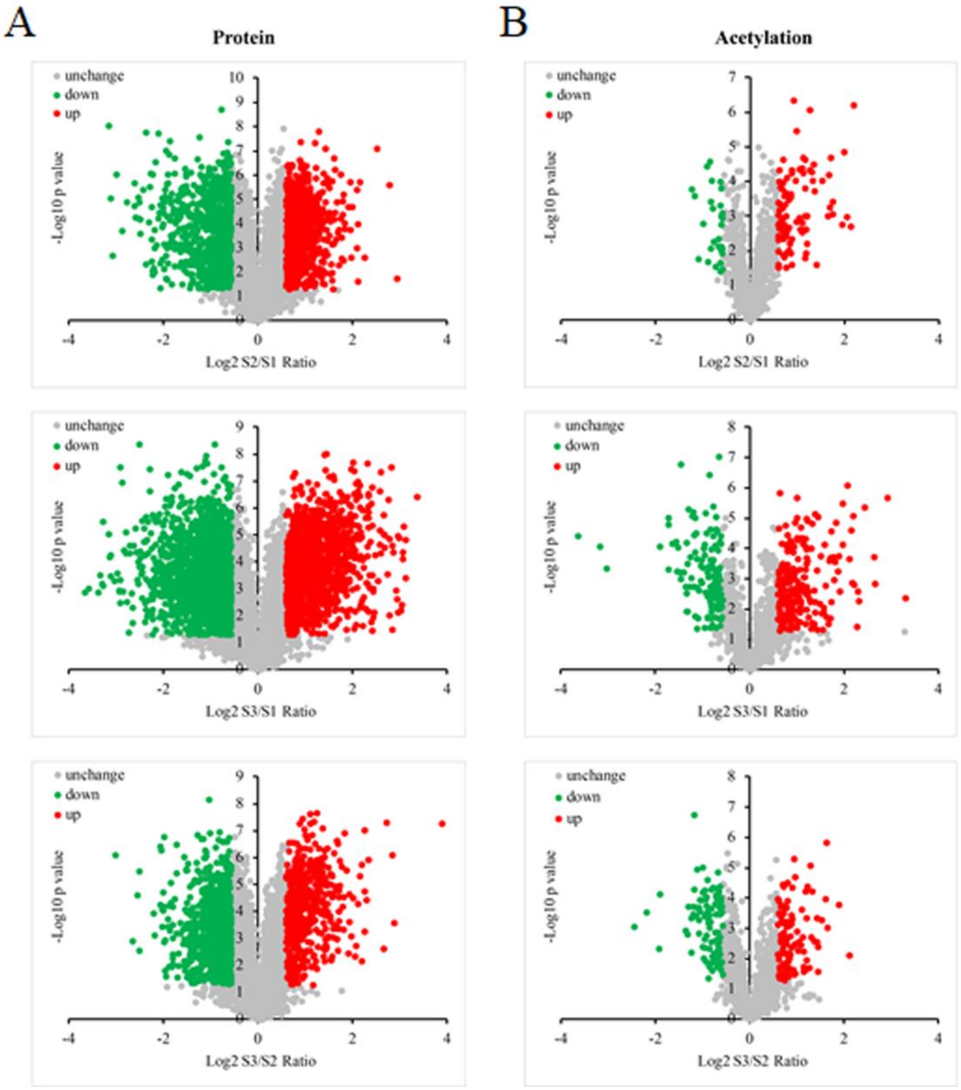

Figure S2. The volcano plots of differential DAPs (A) and ASs (B).

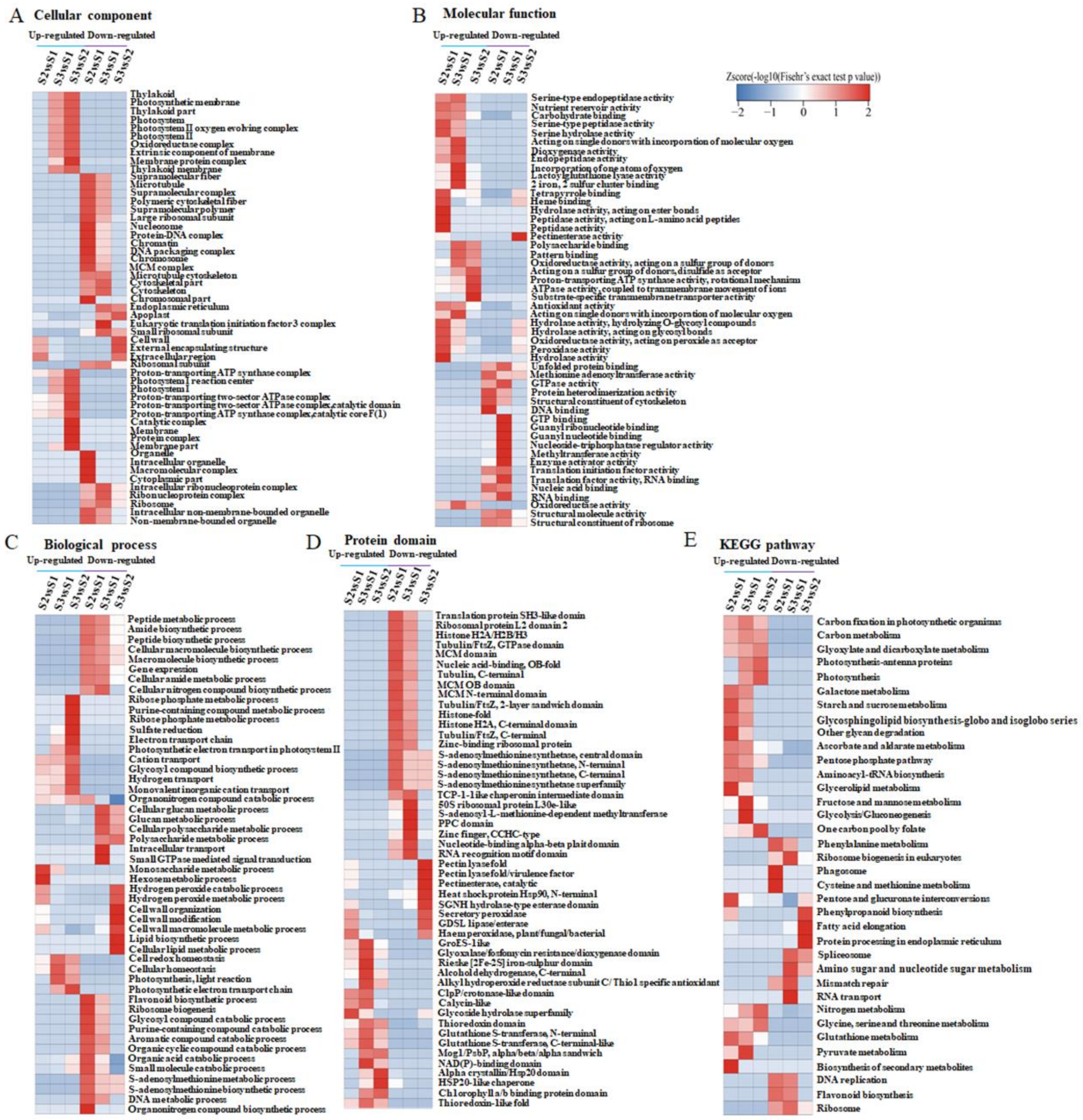

Figure S3. Enrichment-based clustering analysis of DAPs. (A) Cellular component; (B) molecular function; (C) biological process; (D) protein domain; and (E) KEGG pathway.

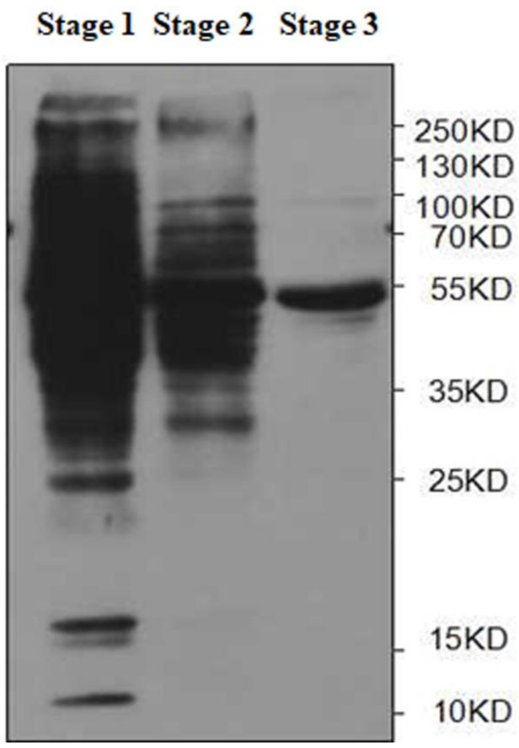

Figure S4. Western blot analysis of acetylation levels at three stages of 'Anji Baicha' leaf development.

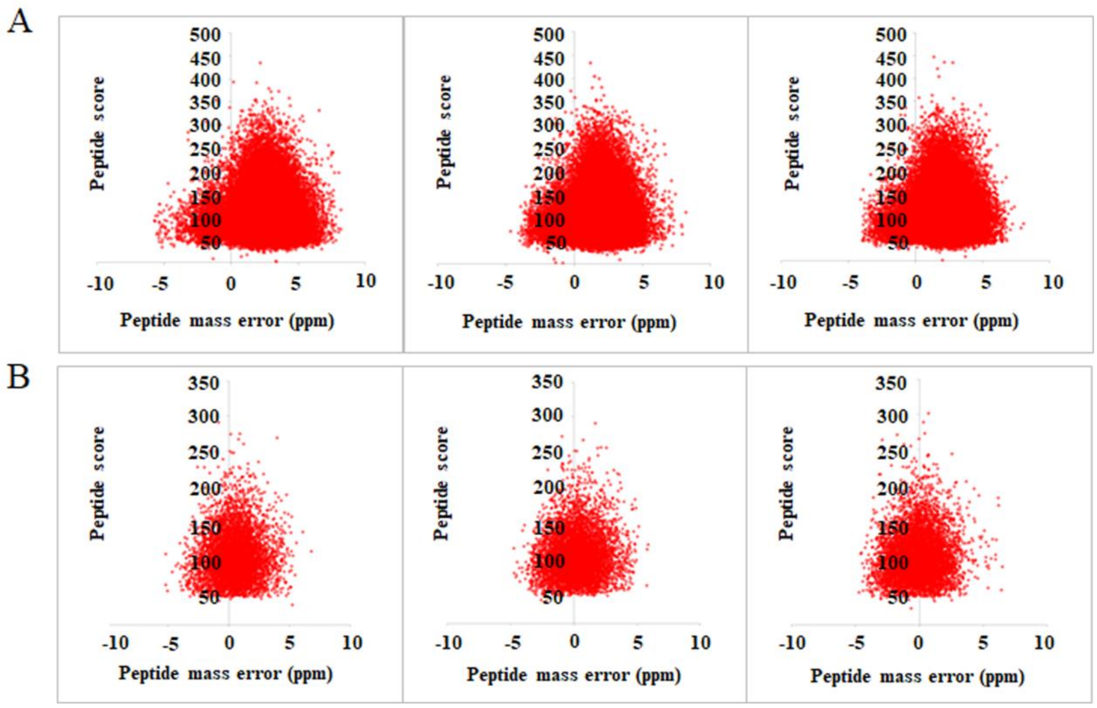

Figure S5. Mass error distribution of peptides identified in the proteome (A) and acetylome (B) profiles (based on three biological replicates).

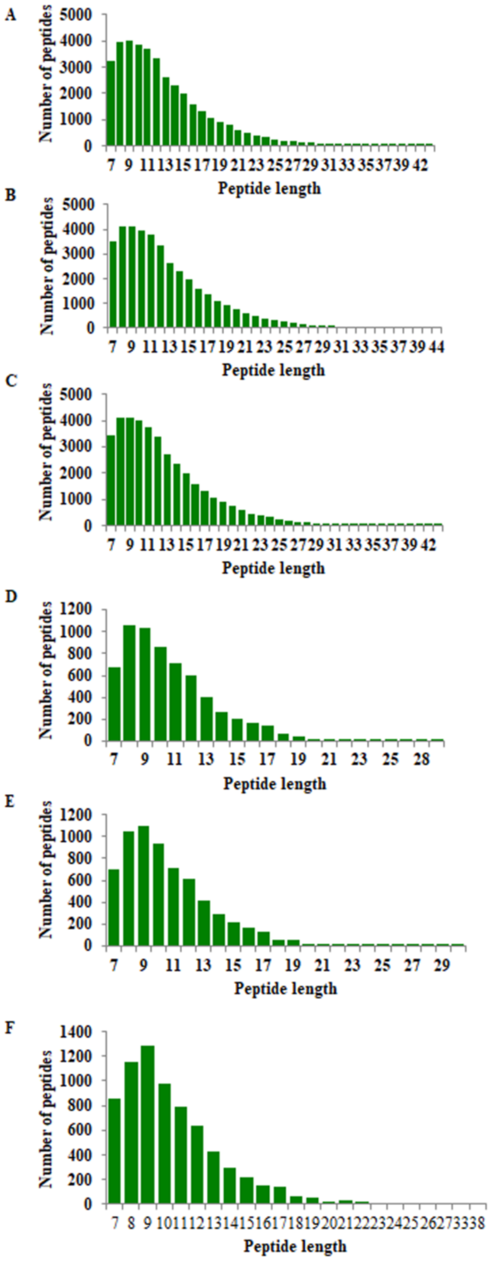

Figure S6. Distribution of peptides in proteome profiles (A–C) and acetylated peptides in acetylome profiles (D–F) according to length (based on three biological replicates).

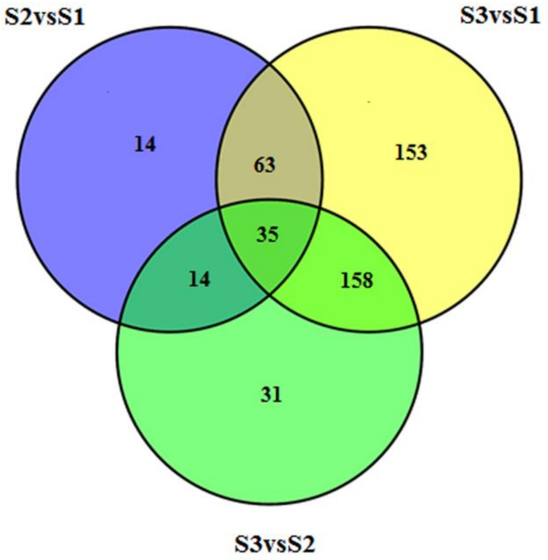

Figure S7. Comparative analysis of differentially ASs among the three 'Anji Baicha' developmental stages.

Supplementary materials and methods

LC−MS/MS measurement and data analysis for complete peptide mixture

The peptide mixture was loaded onto a reversed-phase pre-column (Acclaim PepMap 100, Thermo Scientific). Peptide separation was performed using a reversed-phase analytical column (Acclaim PepMap RSLC, Thermo Scientific). Briefly, the peptide mixture was separated by a linear gradient of 8 to 26% buffer containing 98% acetonitrile and 0.1% formic acid for 22 min, 26 to 40% for 12 min and increasing to 80% in 3 min then holding at 80% for the last 3 min. The flow rate was 400 nl/min. The results were analyzed by Q ExactiveTM Plus hybrid quadrupole-Orbitrap mass spectrometer (Thermo Fisher Scientific). The peptides were subjected to NSI source followed by tandem mass spectrometry (MS/MS) in Q ExactiveTM Plus (Thermo) coupled online to the UPLC. Intact peptides were acquired at a resolution of 70,000. Peptides were selected for MS/MS using the NCE setting as 30, and ion fragments were detected at a resolution of 17,500. A data-dependent "top 20" method was applied to obtain the most abundant precursor ions (mass range 350−1800 m/z) above a threshold ion count of 1E4 in the MS survey scan with 30.0-s dynamic exclusion. The electrospray voltage used was 2.0 kV.

The resulting MS/MS data was processed using MaxQuant with integrated Andromeda search engine (v.1.5.2.8). The tandem MS data were searched against the *C. sinensis* genome dataset (Xia et al., 2017) concatenated with a reverse decoy database. Trypsin/P was specified as the cleavage enzyme allowing up to 2 missing cleavages. The mass error was set to 10 ppm for precursor ions and 0.02 Da for fragment ions. Carbamido methylation on cysteine was specified as a fixed modification. Oxidation on methionine was set as variable modification. False discovery rate (FDR) thresholds for peptide, protein were specified at 1%. The

minimum peptide length was set to 7. All other parameters were set to the default values specified by MaxQuant.
